# Multi-decadal dynamics of ecosystem service interactions in boreal forests (NE China): trade-off synergy strength, drivers, and thresholds

**DOI:** 10.3389/fpls.2026.1921235

**Published:** 2026-08-24

**Authors:** Xueqing Wu, Wei Lu, Lingbo Dong

**Affiliations:** 1 College of Forestry, Northeast Forestry University, Harbin, China; 2 Key Laboratory of Sustainable Forest Ecosystem Management, Ministry of Education, School of Forestry, Northeast Forestry University, Harbin, China; 3 College of Forestry, Hebei Agricultural University, Baoding, Hebei, China

**Keywords:** driving factor, ecosystem service bundle, forest ecosystem services, trade-offs and synergies, XGBoost-SHAP

## Abstract

Quantifying long-term trade-offs and synergies among forest ecosystem services (ESs) is essential for adaptive management, yet the strength and drivers of these interactions over long timescales remain poorly understood. Seven key ESs in the Great Khingan Mountains (GKM), northeast China, were assessed from 1990 to 2024 using multi-source remote sensing data, including water yield (WY), soil conservation (SC), habitat quality (HQ), leisure and recreation (LR), carbon fixation and oxygen release (CF), climate regulation (CR), and net primary productivity (NPP). A Trade-off–Synergy Strength (TSS) metric was developed to characterize both the direction and composite magnitude of pairwise ES co-change. SHAP, and self-organizing maps were integrated to identify dominant drivers and ES bundles. Results showed that SC, CF, CR, and NPP generally increased, whereas WY declined; HQ decreased markedly during 2020–2024, and LR peaked in 2015 before declining. ES interactions were mainly synergy-dominated, although LR-related pairs exhibited persistent trade-offs. Climatic variables accounted for 52.1% of the mean absolute SHAP contribution to TSS variation, while topographic factors mainly shaped ES bundle organization. Under the climatic-change conditions examined in the GKM, SHAP-based analyses suggested that positive TSS responses were generally associated with radiation, precipitation, and temperature growth rates ranging from −8.00 to 5.59 MJ·m^-2^·yr^-1^, −0.74 to 0.03 mm·yr^-1^, and −3.22 to 1.74 °C·yr^-1^, respectively. These findings provide scientific support for climate-adaptive forest management.

## 1 Introduction

Forests are key terrestrial ecosystems that support ecological security, regulate biogeochemical cycles, and provide essential benefits for human well-being and sustainable development (Pan et al., 2024; Zou et al., 2025). Covering nearly one-third of the Earth’s terrestrial surface, forests contribute greatly to global productivity and supply a wide range of ecosystem services (ESs), such as carbon fixation and oxygen release (CF), soil conservation (SC), and water conservation (Bonan, 2008; FAO, 2020; Minkkinen et al., 2020). These services are closely linked and together maintain ecosystem multifunctionality across local, regional, and global scales (Li et al., 2017; Mengjuan et al., 2025). However, climate change and increasing human disturbance are changing forest structure, ecological processes, and service supply, especially in alpine and boreal regions of the Northern Hemisphere where ecosystems are highly sensitive to environmental change (Engert et al., 2024; Pratzer et al., 2026; Xu et al., 2025b). Importantly, these pressures do not affect ESs in isolation. They may also change the relationships among ESs, leading to stronger synergies in some cases and sharper trade-offs in others. Therefore, assessing only individual ESs is not sufficient for understanding forest ecosystem change. A clearer understanding of ES interactions is needed to support ecological restoration, adaptive management, and sustainable forest governance under global change (Peng et al., 2023; Xu et al., 2025a).

Trade-offs and synergies among ESs have become a central topic in ecosystem service research because they directly affect ecosystem multifunctionality and management outcomes (Shoemaker et al., 2019; Zhang et al., 2023a). Synergies occur when multiple ESs increase together, whereas trade-offs arise when the increase of one service is linked to the decline of another. Identifying these relationships is important because forest management often seeks to improve multiple ESs at the same time, but gains in one service may create losses in others. Previous studies have commonly used correlation analysis, regression models, trade-off–synergy indices, bivariate spatial association methods, and ecosystem service multifunctionality metrics to analyze relationships among ecosystem services. Correlation analysis, including Pearson or Spearman correlation, is widely used to identify whether two services show positive or negative statistical associations at the regional scale (Lyu et al., 2019; Spearman, 1904). However, correlation-based approaches often provide an overall statistical relationship and may overlook local spatial heterogeneity, spatial autocorrelation, and temporal changes in composite magnitude of ES co-change (Shaikh et al., 2021). Existing trade-off–synergy indices, such as the trade-off–synergy criterion and trade-off–synergy index proposed by Xue et al. (2023), provide useful tools for identifying pairwise ecosystem service relationships, but relationship direction and interaction magnitude are often represented separately. Bivariate spatial association methods, such as local spatial autocorrelation and bivariate Moran’s I, can reveal local co-location patterns and spatial clusters of ecosystem services (Anselin, 1995), but they mainly describe spatial association rather than the signed intensity of temporal co-change between services. Ecosystem service multifunctionality metrics summarize the overall capacity of multiple services and are useful for assessing multifunctional ecosystem performance (Byrnes et al., 2014; Manning et al., 2018), but they may mask pairwise compensation effects and cannot identify which specific service pairs are responsible for trade-offs or synergies.

To address this methodological gap, we proposed a modified Trade-off–Synergy Strength (TSS) framework as a spatially explicit and signed measure of pairwise ecosystem service interactions. TSS combines the direction of co-change with the composite magnitude of temporal change, so that positive values indicate synergies, negative values indicate trade-offs, and larger absolute values indicate greater magnitude of paired ES changes. Compared with correlation-based or aggregate multifunctionality approaches, TSS provides pixel-level information on both the nature and strength of ecosystem service interactions. This makes it possible to compare interaction states across service pairs, periods, and locations, and to use the resulting values as response variables for driver and response-pattern analyses. In addition to measuring composite magnitude of ES co-change, ecosystem service bundles provide a useful way to identify areas with similar combinations of ESs and translate complex ES patterns into management-relevant zones. Self-organizing maps (SOMs) (Appukuttan et al., 2025), as an unsupervised learning method, are well suited for this purpose because they can group areas with similar ES profiles and show the main patterns in multidimensional data.

Understanding the drivers of ES interactions is important because effective management requires knowing not only where trade-offs and synergies occur, but also why they occur (Zhou et al., 2026). ES relationships can be shaped by climate, topography, vegetation conditions, and human activities, and these effects often vary across space (Ren et al., 2024). Previous studies have applied correlation analysis (Valipour Shokouhi et al., 2024), GWR (Ma et al., 2024), GeoDetector, and other statistical approaches to identify the drivers of ES trade-offs, synergies, and bundles across different ecosystems. These studies have shown that climatic and environmental factors, particularly precipitation, vegetation conditions, and elevation, play important roles in shaping ES interactions (Gao et al., 2021; Xu et al., 2025). However, ES interactions may involve nonlinear responses, combined effects among multiple drivers, and threshold effects, which are difficult to capture using traditional statistical methods alone. Machine learning methods provide useful tools for addressing these issues. In particular, Extreme Gradient Boosting (XGBoost) (Recknagel et al., 2025; Wu et al., 2025) can model nonlinear relationships among multiple variables, while Shapley Additive Explanations (SHAP) can help explain model results by estimating the contribution of each driver and identifying possible nonlinear response patterns (Takefuji, 2025a, b). Recent studies have increasingly combined ecosystem status assessment, spatiotemporal dynamics, and explainable machine learning to identify the mechanisms underlying ecosystem change. For example, Cheng et al. (2025) developed a cropland ecosystem health framework integrating organizational structure, ecosystem services, and environmental quality, and used XGBoost-SHAP to reveal the relative effects of climate change and human activities on ecosystem health dynamics. Referring to this analytical framework, this study focuses on forest ES interactions and integrates TSS, SOM, and XGBoost-SHAP to identify pairwise trade-offs and synergies, spatial bundles, dominant drivers, and climatic thresholds.

The Greater Khingan Mountains (GKM) span both the cold and middle-temperate zones and lie within the transitional belt between a mid-latitude monsoon climate and a temperate continental climate. This unique setting creates strong gradients in temperature, precipitation, vegetation, and human disturbance, making the GKM a typical region for examining how forest ESs and their interactions change under both natural and human pressures. The GKM also plays an important role in maintaining ecological security, conserving biodiversity, regulating carbon and water cycles, and supporting climate adaptation in northern China. Therefore, understanding ES trade-offs, synergies, and bundles in this region is important not only for local forest management, but also for improving knowledge of ecosystem multifunctionality in cold-temperate forest regions. However, previous studies have mainly focused on local parts of the GKM or treated the GKM as one component of broader Northeast China (Wang et al., 2024; Xu et al., 2025a). A long-term and region-wide assessment of ES interactions across the whole GKM, including both Inner Mongolia and Heilongjiang Province, is still lacking.

To address these gaps, this study establishes an integrated framework to assess forest ES interactions across the GKM from 1990 to 2024. We address four key questions: (1) How have major forest ESs changed across spatial and temporal? (2) How have the direction and strength of ES trade-offs and synergies evolved over time? (3) What ES bundles can be identified, and how are they spatially distributed across the region? (4) Which factors drive ES interactions and bundles, and how do ES interactions respond nonlinearly to climatic changes? To answer these questions, we integrated ES assessment, the TSS index, SOM analysis, and XGBoost-SHAP driver interpretation. By linking ES dynamics, composite magnitude of ES co-change, service bundles, and driver effects, this study provides support for differentiated forest management in the GKM and contributes to a deeper understanding of ecosystem multifunctionality in cold-temperate forest regions under global change.

## 2 Materials and methods

### 2.1 Study area

**Figure 1:**
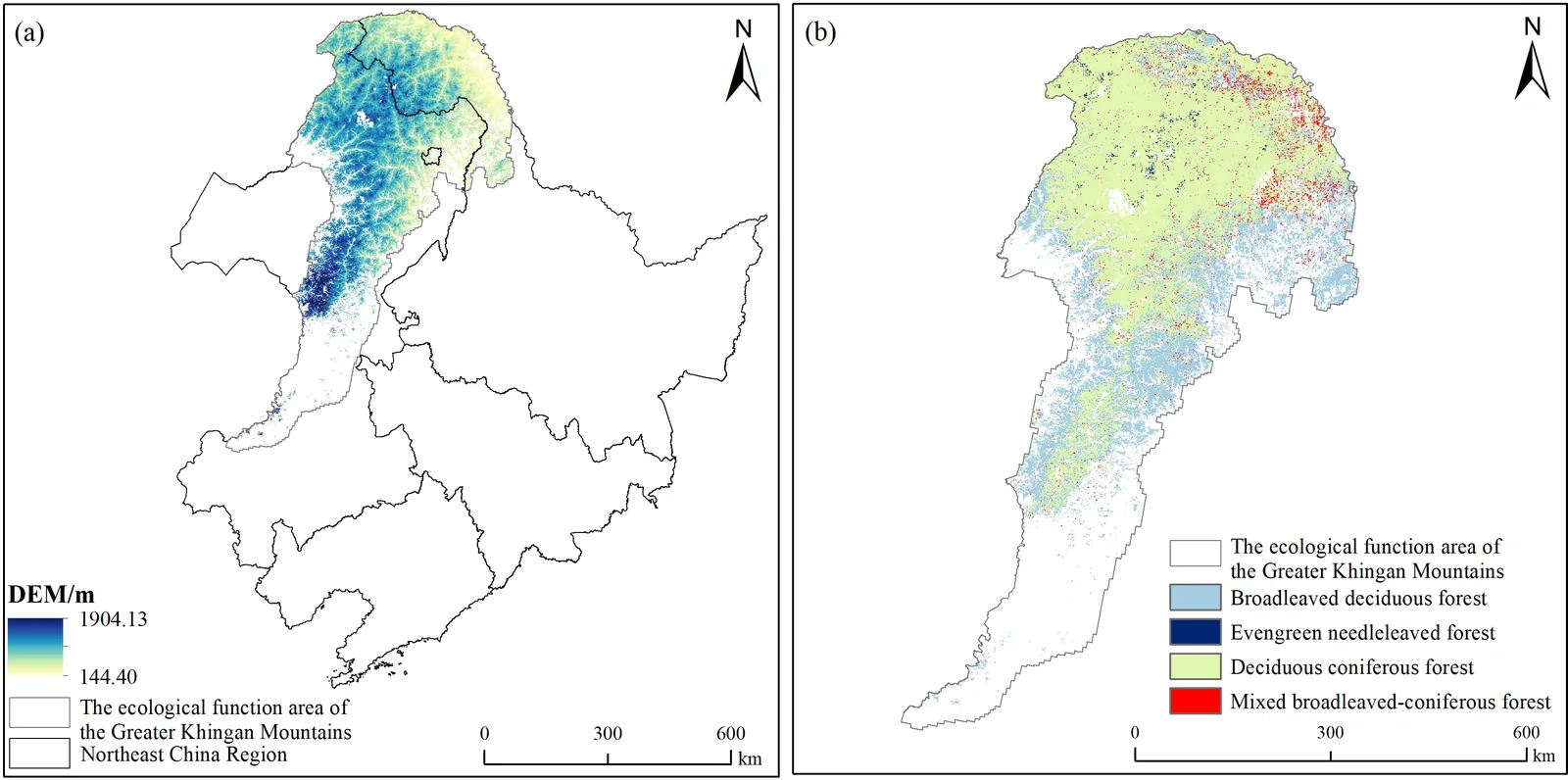
Study area. **(a)** Location of the Great Khingan Mountains in the Northeast China Region; **(b)** Forest types of the Great Khingan Mountains.

The study area is located in northeastern China (**Figure 1**), covering the Inner Mongolia Autonomous Region and Heilongjiang Province (117.74°–126.98°E, 43.80°–53.56°N), which encompasses the entire forested region of the GKM. The region exhibits relatively gentle topographical undulations. Elevations range from approximately 149 to 1900 m. The terrain rises in the southwest and falls in the northeast, marking the northern boundary of China’s second and third topographical tiers. The mean annual temperature is -2.8 °C, with an average annual precipitation of 746 mm. Precipitation decreases from the northeast to the southwest, and snow cover persists for approximately five months annually. The GKM are characterized by typical cold-temperate boreal forests dominated by coniferous, broadleaved, and mixed forest types. The zonal vegetation is mainly composed of Larix gmelinii forests, accompanied by secondary Betula platyphylla and Betula dahurica forests formed after disturbances. Mixed forests commonly include Larix gmelinii, Betula platyphylla, Populus davidiana, and Pinus sylvestris var. mongolica. In moist and high-elevation areas, spruce–fir forests dominated by Picea koraiensis and Abies nephrolepis are also distributed.

### 2.2 Data sources

The research data are divided into two categories: geographical and socio-economic. These datasets are primarily used for ES calculations and XGBoost-SHAP analyses (**Table 1**). Annual NDVI values were generated from monthly Landsat NDVI data using the maximum value composite method. NPP data from different sources were converted to the same unit (g C·m^-2^). To reduce artificial temporal discontinuities between different NPP products, a bias correction was applied before integrating the MOD17A3HGF NPP data into the 1990–2024 NPP series. Because the datasets used in this study differed in temporal coverage and spatial resolution, all data were harmonized before analysis according to their data type and role in ES assessment or driver analysis. All raster datasets were projected to WGS 1984 UTM Zone 51N and resampled to a 1 km × 1 km grid. Categorical data, such as land-cover data, were resampled using the nearest-neighbor method, whereas continuous variables, including climate, NPP, NDVI, population, GDP, and nighttime light intensity, were resampled using bilinear interpolation.

**Table 1:** Data sources and descriptions used in this study.

Category	Data	Period	Resolution	Source
LUCC	ESA CCI Land Cover; NASA MCD12Q1	1990–2020 2024	300 m 500 m	ESA NASA
Climate	Precipitation, temperature, radiation	1951–2024	1 km	China Meteorological Forcing Dataset v2.0 provided by the National Tibetan Plateau/Third Pole Environment Data Center
Potential evapotranspiration	Monthly PET	1901–2024	1 km	Global Change Science Research Data Publication System
Topography	DEM	—	1 km	Copernicus DEM via Google Earth Engine
Soil	Soil texture	—	1 km	HWSD Chinese soil dataset
Vegetation	NDVI	1990–2024	30 m	NASA Landsat series
Productivity	NPP	1985–2015 2016-2024	1 km; 500 m	Global Change Science Research Data Publication System; NASA MOD17A3HGF
Road	Motorways and county roads	—	Vector	OpenStreetMap
Nighttime light	NLI	1984–2020	1 km	National Tibetan Plateau/Third Pole Environment Data Center
Population	POP	1995, 2000, 2005, 2010, 2015, 2019, 2020	1 km	Resource and Environment Science Data Platform, CAS
Economy	GDP	1995, 2000, 2005, 2010, 2015, 2019, 2020	1 km	Resource and Environment Science Data Platform, CAS

For land-cover data, although the ESA CCI product provides annual records, ES calculations were conducted only for the selected assessment years, namely 1990, 1995, 2000, 2005, 2010, 2015, 2020, and 2024. The annual ESA CCI data were mainly used to construct a long-term forest intersection mask to ensure that the analysis focused on pixels that consistently remained forested during the study period. For POP, NLI and GDP, the latest publicly available raster products from the data sources used in this study were from 2020. Therefore, the 2020 POP and GDP data were used as the latest available socioeconomic background for the 2024 LR assessment, rather than being extrapolated to 2024 without direct data support. Unlike WY, SC, NPP, CF, and CR, which require stage-specific climate or biophysical inputs, LR does not require annual climate data for each assessment period. In the XGBoost–SHAP driver analysis, time-series explanatory variables were represented by their multi-year mean values as input factors. This treatment ensured consistency among drivers with different temporal coverages and avoided unsupported extrapolation beyond the available datasets.

### 2.3 Methods

This study was structured into four main steps. First, multi-source remote sensing and environmental data were used to quantify seven indicators of forest ecosystem service supply, regulation, support, and cultural benefits, including water yield (WY), soil conservation (SC), habitat quality (HQ), carbon fixation and oxygen release (CF), climate regulation (CR), leisure and recreation (LR), and net primary productivity (NPP). These indicators were selected to reflect the main ecological functions and human benefits provided by forest ecosystems in the GKM. Second, a TSS metric was developed to characterize the direction and strength of interactions among ESs. Third, SOM analysis was used to identify areas with similar ES profiles and delineate ES bundles. Finally, XGBoost was used to identify the main drivers of TSS and ES bundles, while SHAP was applied to explain driver contributions and detect possible threshold effects. Together, these steps formed an analytical workflow for assessing ES patterns, ES interactions, service bundles, and their driving factors.

#### 2.3.1 ES evaluation

Based on the Millennium Ecosystem Assessment (MEA) classification and the ecological features of the GKM, this study selected seven indicators to represent forest ecosystem service capacity. WY was used to represent provisioning service capacity related to water supply. SC, CF, and CR were selected as regulating services, reflecting soil retention, gas regulation, and climate regulation functions. HQ and NPP were used to represent supporting service capacity, as they indicate biodiversity support, vegetation productivity, and the ecological basis for other services. LR was selected as a cultural service, reflecting the non-material benefits provided by forest landscapes. Together, these indicators describe forest ES capacity from the perspectives of supply, regulation, ecological support, and cultural benefits. WY, SC and HQ were calculated based on the InVest model (Wu et al., 2021), while CF and CR were derived from the Qop (Shang, 2024), AHM (Li et al., 2025). LR was calculated based on the recreational index which developed in this study to evaluate LR services in the GKM, drawing on the framework of Paracchini et al. (2014).To evaluate the sensitivity of the LR index to its individual components, a leave-one-component-out analysis was conducted by separately excluding the GDP-based purchasing-power, population-density, and road-proximity components. Detailed methods and results are provided in **Supplementary Table 1**; NPP data was sourced from publicly available data. Although CF was calculated using NPP as a basic input, CF and NPP were not treated as ecologically identical indicators in this study. NPP represents vegetation productivity, expressed as carbon fixed per unit area, whereas CF represents the carbon fixation and oxygen release service after unit conversion, area-based scaling, and functional coefficient conversion. Specifically, NPP was first converted from g C·m^-2^ to kg C·m^-2^ and then scaled to the 1 km × 1 km evaluation unit to quantify the amount of carbon fixation and oxygen release provided by each evaluation unit. Therefore, the CF–NPP relationship reflects a close linkage between vegetation productivity and carbon-related regulating service, rather than a simple duplication of the same ecological indicator. Specific methodologies and parameters are detailed in the **Supplementary**.

#### 2.3.2 Quantifying trade-offs and synergies

TSS was designed to overcome a specific limitation of existing ecosystem service interaction metrics. Correlation analysis summarizes the average statistical association between two services across all pixels, but it does not produce a spatially explicit map of the direction and magnitude of local temporal co-change. Bivariate spatial association methods can identify local co-location patterns, but they mainly describe spatial clustering rather than the magnitude of temporal co-change. Ecosystem service multifunctionality metrics aggregate several services into a single composite value, which is useful for assessing overall multifunctional capacity but may conceal pairwise conflicts among services. In contrast, TSS focuses on pairwise temporal co-change at the pixel scale and assigns each pixel a signed value representing the direction and composite magnitude of change in a specific ES pair. In this study, the term “strength” refers to the magnitude of paired temporal ES changes represented by TSS, rather than the causal strength of ecological interactions.

Specifically, the directional component of TSS was based on the trade-off–synergy criterion proposed by Xue et al. (2023), which identifies whether two services change in the same or opposite directions. The original intensity component was based primarily on the similarity between the absolute change magnitudes of the two services. However, this formulation could assign a high intensity value when two services showed similar but very small changes. It could also assign a low value to an asymmetric response in which one service changed substantially while the other changed only slightly, although such a response may still be ecologically or managerially important. Therefore, in this study, the TSC and its directional reclassification were retained, whereas the TSI was reformulated to represent the composite magnitude of temporal change in the paired services. A separate asymmetry index was further introduced to distinguish relatively balanced changes from responses dominated by one service. The complete TSS framework, including the directional criterion, composite change magnitude, and asymmetry index, is defined in Equations 1–5.

Before calculating TSS, all ES values were normalized to a common 0–1 scale using the min–max normalization method to remove the effects of different units and value ranges. Raw ES values were used for ES assessment and descriptive analysis, whereas normalized ES values were used for TSS calculation. In the following equations, ES refers to the normalized ES value. The temporal change of each ES was calculated as the difference between the normalized values at two time points, namely the value at *t_2_* minus the value at *t_1_*. Positive changes indicate an increase in service capacity, whereas negative changes indicate a decrease. In this study, temporal changes were calculated for both adjacent periods and the overall period from 1990 to 2024.

Since TSC only indicates the direction of the relationship, we reclassified its value to represent the relationship type. Specifically, positive TSC values were assigned a value of 1, and negative TSC values were assigned a value of −1. When either ES showed zero or near-zero temporal change, the reclassified TSC value was assigned as 0 and interpreted as no clear trade-off or synergy. This reclassified TSC was subsequently combined with the revised TSI to construct the TSS index, allowing the final metric to represent both the direction and composite magnitude of pairwise ES changes.

(1) $$TSC=\frac{ES_{i1t2}-ES_{i1t1}}{ES_{j1t2}-ES_{j1t1}}$$

(2) $$TSC'=\{\begin{array}{c} 1,TSC>0\\ -1,TSC<0 \end{array}$$

where TSC is the trade-off synergy criterion, while *ES_i1t2_* and *ES_i1t1_* represent ecosystem service type i during time periods t_2_ and t_1_, respectively. Similarly, *ES_j1t2_* and *ES_j1t1_* represent ecosystem service type j during time periods t_2_ and t_1_, respectively. If TSC > 0, this indicates that the paired ES_i_ and ES_j_ change in the same direction, signifying a synergistic relationship. Conversely, if TSC < 0, this denotes a trade-off relationship.

(3) $$TSI=\frac{\left|\left|\Delta ES_{i}\right|+\left|\Delta ES_{j}\right|\right|}{2}$$

The revised TSI was calculated as the arithmetic mean of the absolute temporal changes in the paired services. It ranges from 0 to 1 and represents the composite magnitude of temporal change in the ES pair. A value close to 0 indicates that both services changed only slightly, whereas a larger value indicates a greater overall magnitude of change across the two services. Because the arithmetic mean incorporates the absolute changes of both services, an asymmetric response in which one service changes substantially while the other changes only slightly can still contribute to the composite magnitude, although its imbalance is further evaluated using the asymmetry index. However, a high TSI does not necessarily indicate that the two services changed by similar magnitudes; the balance between their changes was evaluated separately using the asymmetry index.

(4) TSS=TSC'×TSI

TSS ranges from −1 to 1. Positive values indicate same-direction changes and were operationally classified as synergies, whereas negative values indicate opposite-direction changes and were classified as trade-offs. The absolute TSS value represents the mean absolute magnitude of temporal change in the paired services. A larger positive TSS therefore indicates a greater same-direction composite change, whereas a larger absolute negative TSS indicates a greater opposite-direction composite change. In this study, “strength” refers to the composite magnitude of temporal change in the paired services rather than the strength of a causal ecological interaction between them. However, because the composite magnitude represented by TSS does not indicate whether the two services contributed equally to the paired response, an additional asymmetry index was calculated.

To complement TSS, we further introduced an asymmetry index (ASI) to quantify the imbalance between the absolute temporal changes in the two services:

(5) $$ASI=\{\begin{array}{c} \frac{\left|\left|\Delta ES_{i}\right|-\left|\Delta ES_{j}\right|\right|}{\left|\left|\Delta ES_{i}\right|+\left|\Delta ES_{j}\right|\right|}\\ 0 \end{array}\begin{array}{c} ,\left|\Delta ES_{i}\right|+\left|\Delta ES_{j}\right|>0\\ ,\left|\Delta ES_{i}\right|+\left|\Delta ES_{j}\right|=0 \end{array}$$

where *ASI* ranges from 0 to 1. Values close to 0 indicate that the two services changed by similar absolute magnitudes, whereas values close to 1 indicate a highly asymmetric response in which the temporal change was primarily dominated by one service. ASI does not indicate the direction of the relationship or the overall magnitude of the paired changes. Instead, it provides complementary information for distinguishing relatively balanced ES changes from responses dominated by one service. When one service showed zero or near-zero change while the other changed noticeably, *TSC’* remained 0 according to the directional classification rule, whereas ASI approached 1, indicating a highly asymmetric response without a clearly identifiable trade-off or synergy.

TSS and ASI were therefore interpreted jointly. A large absolute TSS combined with a low ASI indicates a relatively large and balanced paired response, whereas a large absolute TSS combined with a high ASI indicates that the composite change was mainly driven by one service. A low absolute TSS and a low ASI indicate that both services changed only slightly, while a high ASI highlights potentially relevant asymmetric responses that may require further interpretation, particularly when the service showing the larger change was declining or represented a critical ecological or socioeconomic function.

To evaluate the robustness of the TSS framework, we conducted a series of sensitivity analyses from multiple perspectives. First, the dominant interaction directions identified by TSS were compared with Pearson, Spearman, and partial correlation analyses. Second, threshold sensitivity analyses were performed using different absolute TSS thresholds (|TSS| > 0, 0.05, 0.10, 0.15, and 0.20) to examine whether interaction classifications were sensitive to threshold selection. Third, because CF was calculated using NPP as a basic input, a sensitivity analysis excluding the CF–NPP pair was conducted to determine whether the dominant ES relationship patterns were driven by this potentially dependent relationship. Finally, bootstrap perturbation and leave-one-pair-out analyses were performed to evaluate the structural stability of the identified ES interaction patterns. Detailed robustness assessment results are provided in **Supplementary Material** (**Supplementary Tables 2**-**S6**). These comparisons were used only to assess consistency in interaction direction, because correlation-based methods do not quantify the composite magnitude represented by TSS.

The aforementioned method can only explore pairwise relationships between ecosystem services. To further investigate the spatial clustering characteristics of ES, SOM analysis was used to identify areas with similar ES profiles and delineate ES bundles (Park and Kim, 2026; Shen et al., 2021). A total of 118,360 valid pixels were identified, and six service bundles were delineated using the “kohonen” package in R 4.4.3.

#### 2.3.3 Extreme gradient boosting model

Chen and Guestrin (2016) proposed an ensemble learning algorithm that trains new base models to correct the errors of preceding models, thereby progressively reducing the overall bias of the additive model. This approach falls within the category of gradient boosting tree algorithms. The XGBoost model has gained widespread popularity due to its high computational efficiency, which simplifies the search for optimal solutions and accelerates computation. XGBoost is widely regarded as one of the fastest and most robust decision tree algorithms, capable of addressing a wide range of regression and classification problems (Ma et al., 2021).

This study selected five categories of factors—meteorological, topographical, socio-economic, soil, and vegetation—from which the following input variables influencing trade-offs and synergies were chosen: precipitation growth rate, temperature growth rate, radiation growth rate, digital elevation model (DEM), slope, nighttime light index (NLI), population density (POP), soil pH (pH), normalized difference vegetation index (NDVI), and stand age (SA). To assess multicollinearity among factors, pixels were randomly sampled from each raster variable, and Pearson correlation coefficients and variance inflation factors (VIFs) were calculated. The VIF values ranged from 1.05 to 1.49, all below the commonly used threshold of 5, indicating no substantial multicollinearity among the independent variables.

The 21 pairwise TSS values and the six SOM-derived service bundle classes were used as dependent variables in separate XGBoost models. The training and testing datasets were split in an 8:2 ratio. Bayesian parameter tuning was employed to optimize the models using the ‘xgboost’ package in R 4.4.3. The optimized model parameters and predictive performance of the 21 pairwise TSS models are summarized in **Supplementary Table 7**.

#### 2.3.4 SHAP interpretability analysis

To identify the thresholds that influence trade-offs and synergies, and to understand variations in service bundles, the SHAP method was introduced to address the “black box” nature of XGBoost models. Based on the Shapley value concept in game theory, SHAP quantifies each feature’s contribution to the model and explains prediction outcomes (Pelegrina et al., 2023). In this study, the ‘SHAPforxgboost’ implementation in R 4.4.3 was employed to identify the key drivers of trade-offs and synergies and service bundle characteristics. SHAP’s feature dependence analysis was used to identify nonlinear response patterns and model-derived response ranges of the influencing factors.

## 3 Results

### 3.1 Spatial patterns of forest ecosystem services

**Figure 2:**
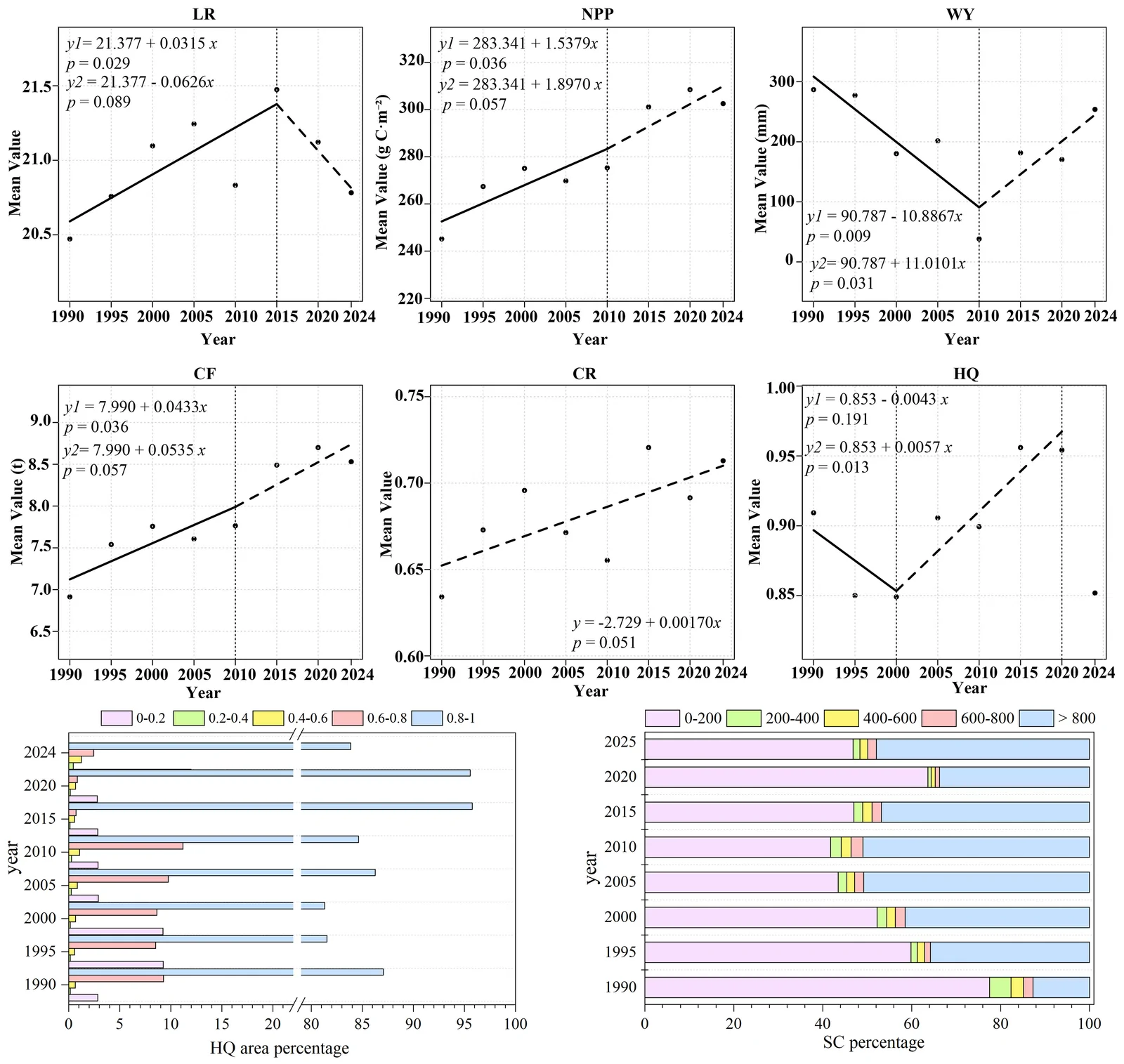
Changes in ESs between 1990 and 2024. The dashed vertical lines indicate the breakpoint years of the segmented trends (WY, water yield; SC, soil conservation; HQ, habitat quality; CF, carbon fixation and oxygen release; CR, climate regulation; LR, leisure and recreation; NPP, net primary productivity.).

The seven ecosystem services in the GKM exhibited clear differences in temporal trends and spatial patterns across the eight study periods (**Supplementary Figure 1**). Overall, most ESs were higher in 2024 than in 1990, including SC, NPP, CF, CR, and LR, whereas WY and HQ were lower than their initial levels. However, these changes were not linear, and several services showed clear stage-specific fluctuations rather than continuous improvement (**Figure 2**). WY decreased sharply before 2010 and then partially recovered after 2010, indicating strong temporal variation in water yield. NPP and CF showed similar temporal patterns, increasing slowly before 2010, rising markedly during 2010–2020, and then slightly declining in 2024, while remaining higher than their initial levels. SC increased rapidly during 1990–2005, declined during 2010–2020, and rebounded in 2024. Results showed that SC, CF, CR, and NPP generally increased, WY declined, HQ decreased markedly between 2020 and 2024, and LR peaked in 2015 before declining. CR fluctuated over time but remained higher in 2024 than in 1990. LR fluctuated slightly, reached its highest value in 2015, and then declined during 2015–2024. The leave-one-component-out sensitivity analysis showed that the broad spatial pattern of LR remained stable after excluding individual socioeconomic or accessibility components. However, the timing of the regional maximum was sensitive to the GDP-based purchasing-power component: the peak remained in 2015 after excluding population density or road proximity but shifted to 2005 after excluding GDP (**Supplementary Table 1**). These changes indicate that improvements in ESs were not evenly maintained across all services.

**Figure 3:**
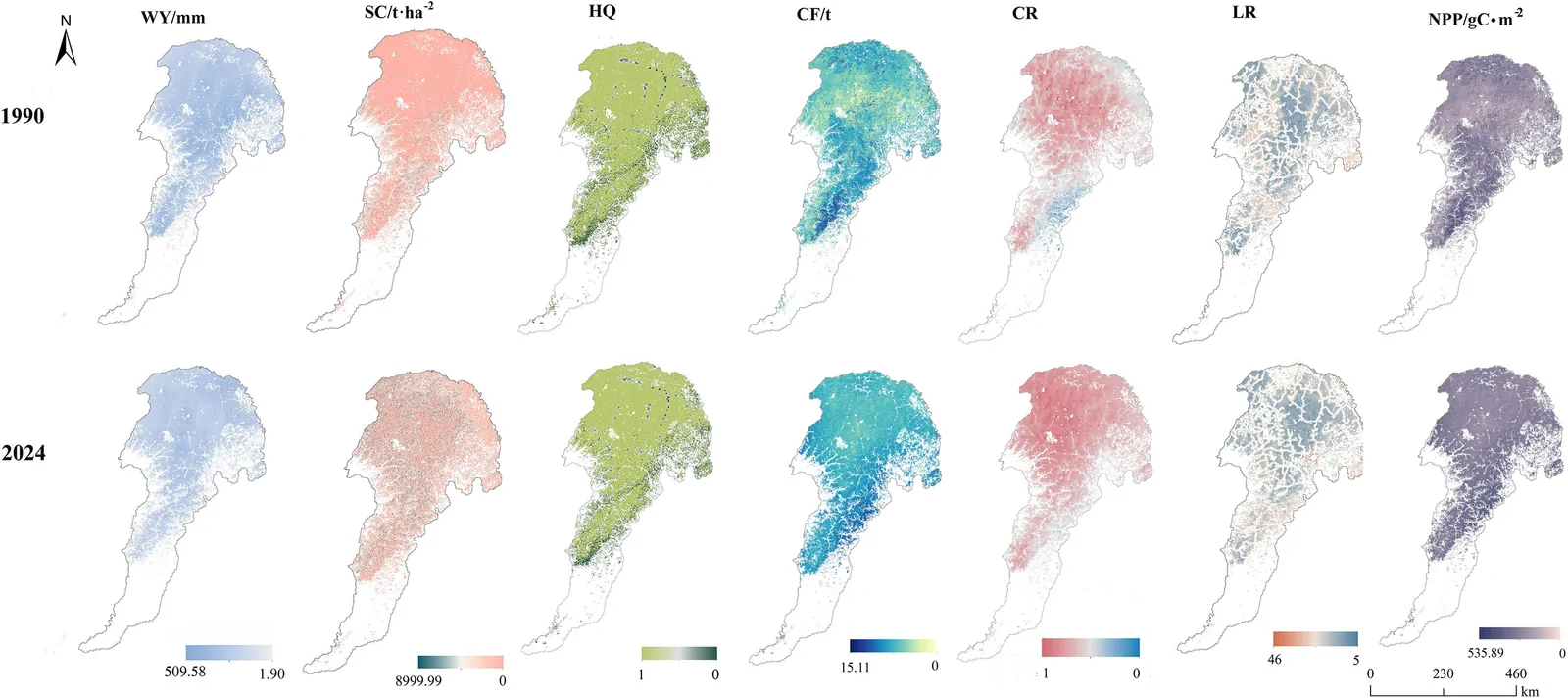
Spatial patterns of the forest ecosystem services in Great Khingan Mountains in 1990 and 2024 (WY, water yield; SC, soil conservation; HQ, habitat quality; CF, carbon fixation and oxygen release; CR, climate regulation; LR, leisure and recreation; NPP, net primary productivity).

In terms of spatial distribution (**Figure 3**), high WY values gradually extended from the southwestern to the northeastern parts of the GKM, while the northwestern region consistently exhibited low values. The overall distribution of SC remained relatively stable, but high-SC areas were more extensive in 2024 than in 1990, despite clear temporal fluctuations. The GKM generally maintained high HQ values, but the proportion of low-HQ areas increased during the study period, mainly in the southeastern part of the region. CF and NPP showed broadly similar spatial patterns across the eight study periods. High values were mainly distributed in the central and southern forested areas of the GKM. Compared with 1990, high-value areas of both services expanded and became more continuous by 2024, indicating the close linkage between vegetation productivity and carbon fixation.

### 3.2 Trade-off and synergy of ESs

**Figure 4:**
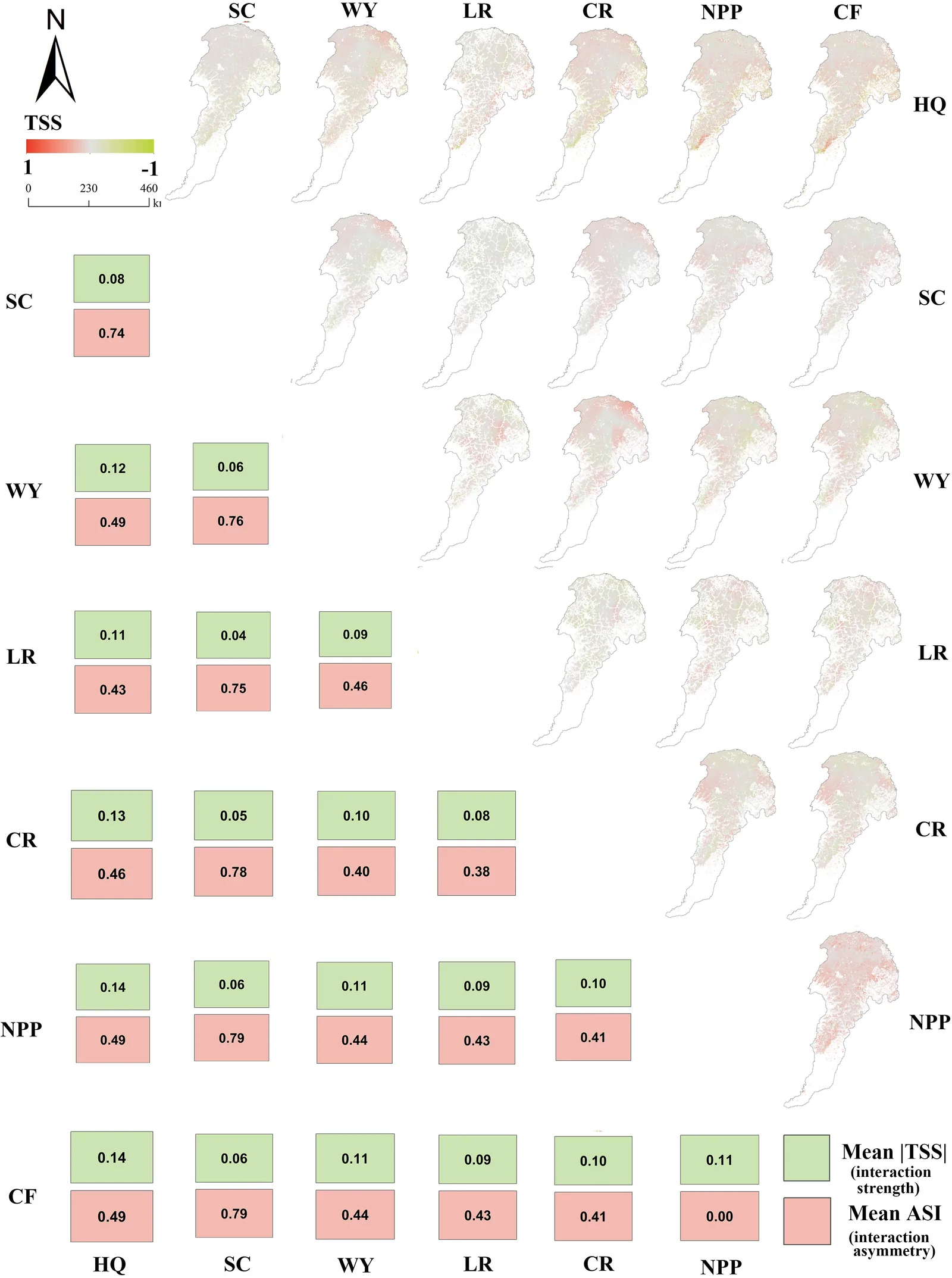
Spatial patterns of pairwise ecosystem service relationships and their average temporal change characteristics during 1990–2024.

The TSS results revealed clear spatial and temporal differences in ES interactions across the GKM. For the overall change from 1990 to 2024, most ES pairs were dominated by synergies rather than trade-offs (**Figure 4**). Among the 21 ES pairs, 15 pairs showed predominantly synergistic relationships. Strong synergies mainly occurred among vegetation, carbon, soil, habitat, and climate-related services, including CF-NPP, SC-CR, SC-NPP, SC-CF, SC-HQ, NPP-HQ, CR-NPP, CR-HQ, CF-CR, and CF-HQ. The synergistic areas of these pairs ranged from 70.9% to 93.8% of the study area, with the largest synergistic area observed for CF–NPP, which is expected because CF was calculated using NPP as a basic input. For comparison, correlation-based approaches showed moderate agreement with TSS-derived interaction directions. Pair-level classifications are provided in **Supplementary Table 2**, and period-level agreement is summarized in **Supplementary Table 3**. Threshold sensitivity analysis further showed that the agreement between TSS- and ASI-derived classifications varied under different absolute TSS thresholds (**Supplementary Table 4**). Because CF was calculated using NPP as a basic input, we examined whether this inherent dependency affected the dominant interaction patterns. After excluding the CF–NPP pair, 87 of the remaining 160 pair-period cases were synergy-dominated, whereas 73 were trade-off-dominated, with mean synergy and trade-off area ratios of 51.81% and 48.19%, respectively (**Supplementary Table 5**). Bootstrap perturbation and leave-one-pair-out analyses further indicated that the dominant interaction structures were generally stable, and excluding any individual ES pair changed the synergy proportion by less than 3% (**Supplementary Table 6**). These results indicate that the TSS-derived interaction patterns were not controlled by a single ES pair, although their agreement with conventional correlation-based classifications was incomplete.

Spatially, these synergies were mainly distributed in the northern and central forest regions. Spatially, synergistic TSS values were mainly distributed in the northern and central forest regions, whereas negative TSS values were mainly associated with LR-related pairs, especially SC–LR, CR–LR, LR–HQ, LR–NPP, and CF–LR. WY-related pairs showed more balanced patterns, indicating stronger spatial variability in water-related ES interactions.

**Figure 4** illustrates the spatial patterns of pairwise ecosystem service relationships during 1990–2024, together with their average composite change magnitude (Mean |TSS|) and temporal change asymmetry (Mean ASI). Overall, ecosystem service relationships exhibited pronounced spatial heterogeneity across the Greater Khingan Mountains. Although both synergistic (positive TSS) and trade-off (negative TSS) patterns were observed, t their spatial distributions differed considerably among ecosystem service pairs, reflecting differences in the direction and composite magnitude of temporal ES changes. Synergistic patterns were mainly concentrated in the central and northeastern forest regions, whereas trade-off patterns occurred more frequently in peripheral areas, indicating substantial spatial variation in the direction and magnitude of ecosystem service co-changes.

The average composite magnitude of temporal change (Mean |TSS|) varied substantially among ecosystem service pairs, ranging from 0.04 to 0.14 (**Figure 4**). The largest composite temporal changes were observed between HQ and NPP (Mean |TSS| = 0.14) and between HQ and CF (Mean |TSS| = 0.14), followed by HQ–CR (0.13) and HQ–WY (0.12), indicating relatively large composite temporal changes in these ecosystem service pairs. Comparatively high composite magnitude of ES co-changes were also identified for WY–CF (0.11), WY–NPP (0.11), LR–HQ (0.11) and CF–NPP (0.11), suggesting relatively large synchronous temporal changes among habitat quality, vegetation productivity, and carbon-related ecosystem services. In contrast, the weakest composite magnitude of ES co-changes occurred between SC and LR (0.04) and between SC and CR (0.05), indicating relatively weak overall interactions involving soil conservation and these ecosystem services.

The degree of temporal change asymmetry showed even greater variation, with Mean ASI ranging from 0.00 to 0.79 (**Figure 4**). The highest asymmetry occurred in SC–NPP (0.79) and SC–CF (0.79), followed by SC–CR (0.78), SC–WY (0.76) and SC–LR (0.75). These results indicate that paired changes involving soil conservation were generally characterized by asymmetric temporal responses, where one ecosystem service contributed more strongly to the observed co-change pattern. Conversely, the interaction between NPP and CF exhibited an almost zero Mean ASI, reflecting their nearly reflecting the expected strong coupling caused by the shared carbon-related basis between NPP and CF. Most of the remaining ecosystem service pairs exhibited moderate asymmetry (approximately 0.38–0.49), suggesting that although differences in response magnitude existed, both ecosystem services contributed to the observed paired temporal changes.

The joint interpretation of Mean |TSS| and Mean ASI revealed that composite magnitude of ES co-change and temporal change asymmetry represented two complementary characteristics of ecosystem service relationships. Ecosystem service pairs with similar composite magnitude of ES co-changes could exhibit markedly different degrees of asymmetry. For example, WY–HQ and HQ–CR both showed relatively strong interactions (Mean |TSS| = 0.12–0.13), while their Mean ASI values remained below 0.50, indicating comparatively balanced responses between paired ecosystem services. In contrast, SC–NPP and SC–CF exhibited only moderate composite magnitude of ES co-changes (Mean |TSS| = 0.06), but their Mean ASI reached 0.79, indicating that these interactions were primarily driven by disproportionate changes in one ecosystem service. These results demonstrate that the magnitude of paired ES changes alone cannot fully characterize ecosystem service relationships, and that incorporating temporal change asymmetry provides additional insight into the balance of temporal responses between paired services.

### 3.3 Spatial distribution of ES bundles

Six ecosystem service bundles were spatially identified using SOM analysis: a key synergetic bundle, characterized primarily by synergistic relationships between HQ and WY; an integrated ecological bundle, featuring interactions between HQ, CR, and WY; an ecological-recreational synergy bundle, maintaining a sustainable equilibrium between ecological and recreational aspects, where HQ, CR, and WY exhibit synergistic relationships; a multi-ecological synergy bundle, demonstrating relationships between all components except HQ; an ecological transition bundle, primarily synergistic between HQ, CR, and NPP, with mild synergies between LR and CF; and a recreational development bundle.

**Figure 5:**
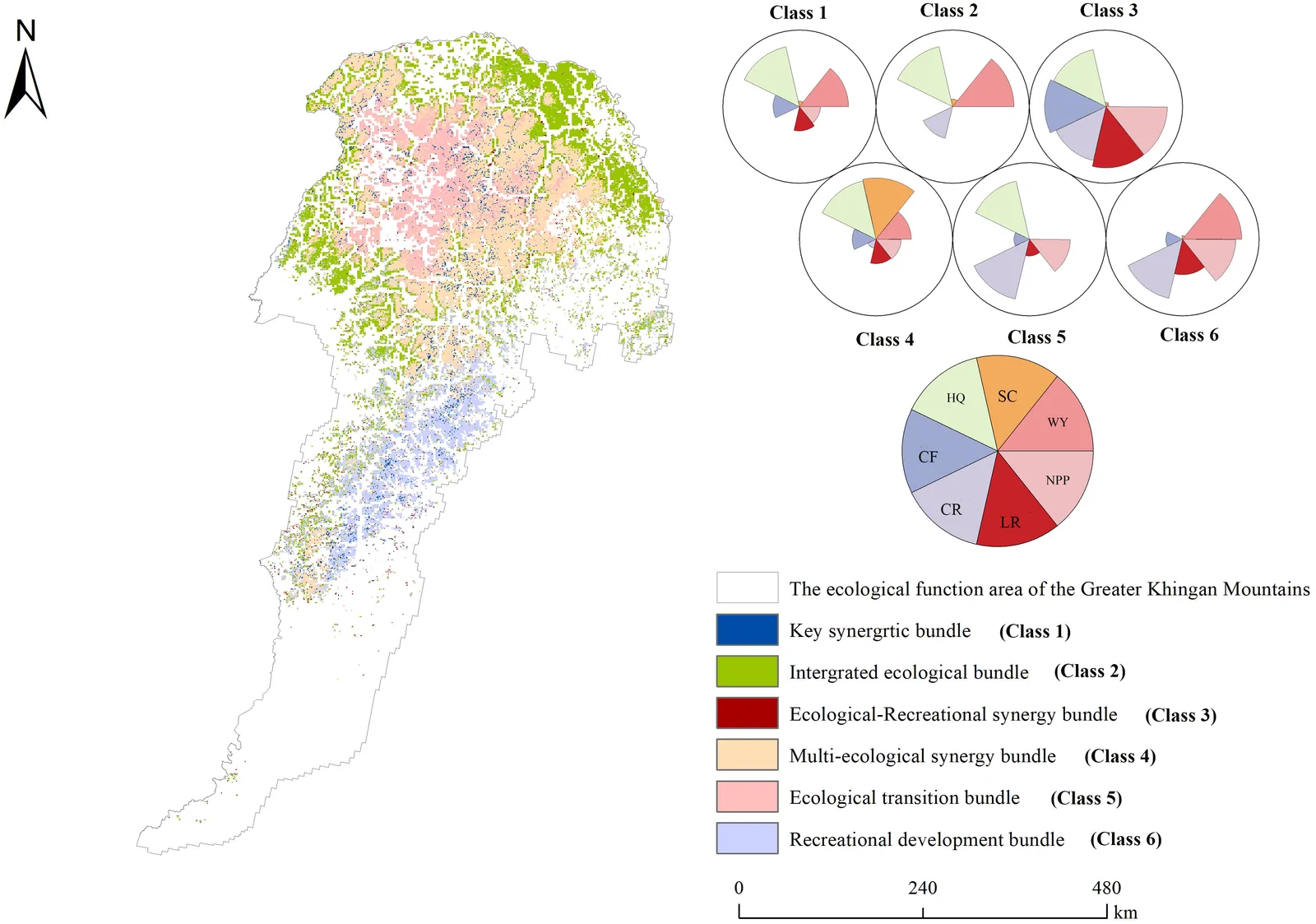
Spatial pattern of forest ecosystem service bundles in Great Khingan Mountains (WY, water yield; SC, soil conservation; HQ, habitat quality; CF, carbon fixation and oxygen release; CR, climate regulation; LR, leisure and recreation; NPP, net primary productivity).

These bundles represent distinct synergistic relationships among different ecosystem services (**Figure 5**). The six bundles, ranked by area proportion from largest to smallest, are: the multi-ecological synergy bundle (30.76%), the integrated ecological bundle (27.54%), the recreational development bundle (19.51%), the ecological transition bundle (17.54%), the key synergetic bundle (3.2%), and the ecological-recreational synergy bundle (1.45%).

The multi-ecological synergy bundle and the ecological transition bundle are predominantly distributed in the central region of the GKM. The integrated ecological bundle is primarily located in the northern and western parts of the GKM, while the recreational development bundle is mainly distributed in the southern region.

### 3.4 Effects of driving factors on the trade-off/synergy relationships

#### 3.4.1 Effects of driving factors on the TSS

XGBoost modeling was used to explain the spatial variation in TSS for the 21 ecosystem service pairs during 1990–2024. The independent test-set R² values ranged from 0.12 to 0.87 (**Table 2**), indicating that model explanatory power varied among service pairs. Most models showed moderate to high predictive performance, although explanatory power varied among ES pairs. Relatively low R² values were observed for several HQ-related relationships, including NPP–HQ, CF–HQ, SC–HQ, and LR–HQ. These lower values suggest that habitat-related ES interactions involve additional ecological processes that were not fully represented by the selected environmental predictors. In contrast, the lower explanatory power of the CF–NPP model is mainly related to the intrinsic coupling between these two services because CF was directly calculated from NPP, resulting in limited independent variation that could be explained by external environmental drivers. Therefore, SHAP interpretations for these low-performance models were treated with caution.

**Table 2:** Predictive performance and number of iterations for the XGBoost models.

ES pair	R^2^	RMSE	n_rounds	max_depth	ES pair	R^2^	RMSE	n_rounds	max_depth
SC-CR	0.87	0.01	860	14	SC-LR	0.68	0.02	1499	13
WY-CR	0.74	0.05	1495	12	SC-HQ	0.17	0.11	107	11
WY-SC	0.79	0.03	860	11	LR-HQ	0.20	0.12	262	15
CR-HQ	0.26	0.13	1500	14	CF-LR	0.5	0.07	325	11
CR-LR	0.50	0.03	1500	13	LR-NPP	0.49	0.07	1498	12
WY-HQ	0.21	0.13	1305	14	SC-NPP	0.46	0.04	857	9
WY-LR	0.62	0.07	347	13	SC-CF	0.59	0.04	418	13
WY-NPP	0.47	0.09	410	14	CF-HQ	0.13	0.15	552	13
WY-CF	0.48	0.09	1190	13	NPP-HQ	0.12	0.15	212	9
CF-CR	0.64	0.06	1431	14	CF-NPP	0.42	0.06	403	15
CR-NPP	0.64	0.06	324	15					

**Figure 6:**
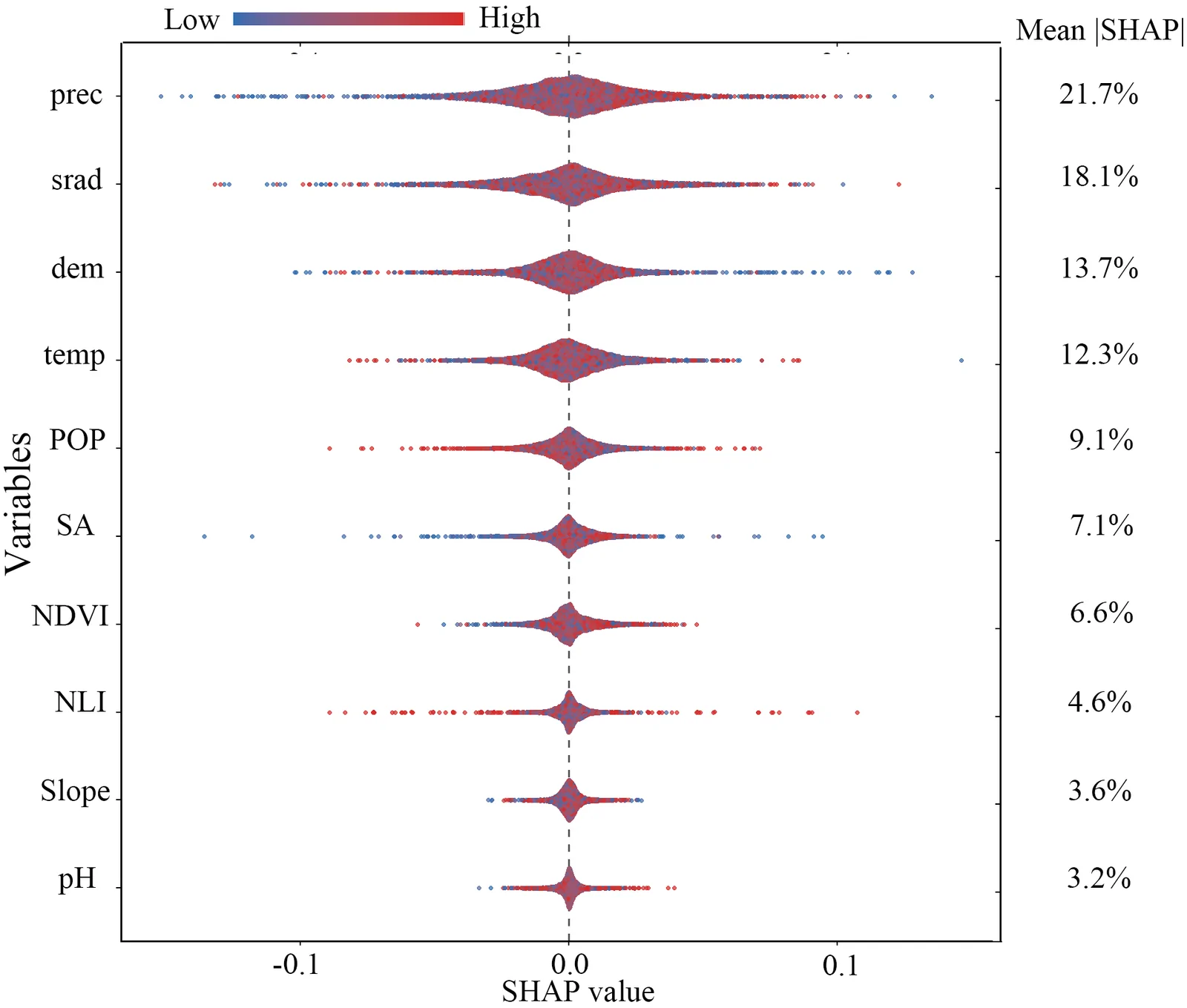
TSS feature importance map (NLI, night light data; pH, soil pH; SA, forest age; POP, population density; prec, precipitation; NDVI, normalized difference vegetation index; temp, temperature growth rate; srad, radiation growth rate; DEM, digital elevation model).

The TSS Feature Importance Map (**Figure 6**) shows that climatic variables, particularly precipitation, radiation, and temperature growth rates, together with topographic factors, were the main drivers of TSS variation. SHAP-based partial dependence analysis revealed nonlinear response patterns of three key factors on TSS (**Supplementary Figure 3**).

Precipitation growth rate showed relatively clear threshold behavior. For many ES pairs, positive SHAP responses occurred when precipitation growth rates were within approximately −0.74 to 0.03 mm·yr^-1^. This suggests that increasing precipitation growth generally promoted synergies among carbon, habitat, soil, water, productivity, and climate-related services. However, some LR-related and climate-related pairs showed positive responses under lower precipitation-growth conditions. For example, CF–LR, LR–NPP, LR–HQ, SC–LR, CR–LR, and WY–CR showed positive SHAP values below specific negative thresholds, while CR–LR, LR–NPP, and LR–HQ also showed positive responses under higher precipitation-growth ranges. These results indicate that the effect of precipitation on TSS was not uniform across ES pairs.

Radiation growth rate showed the most complex response pattern, with several ES pairs exhibiting multiple positive SHAP intervals. Moderate radiation-growth ranges (-8.00 to 5.59 MJ·m^-2^·yr^-1^) promoted synergies for several regulating and supporting service pairs, such as SC–CR, SC–CF, SC–NPP, CF–CR, CR–NPP, and CR–HQ. WY-related relationships also responded positively within both low and high radiation-growth ranges. For example, WY–CF, WY–NPP, WY–SC, WY–CR, and WY–HQ showed positive responses under very low radiation growth, while several of these pairs also showed positive responses when radiation growth exceeded approximately 3.54 to 7.06 MJ·m^-2^·yr^-1^. These results suggest that radiation influenced ES interactions through multiple response ranges rather than through a single threshold.

Temperature growth rate mainly promoted synergies within moderate negative to near zero ranges, although several ES pairs also showed positive responses under extremely low or high temperature-growth conditions. For example, CF–CR, CR–NPP, CR–HQ, SC–CR, SC–HQ, WY–HQ, WY–CR, and WY–SC showed positive SHAP responses within ranges from approximately -3.22 to 1.74 °C·yr^-1^, LR-related pairs, including CF–LR, LR–NPP, LR–HQ, SC–LR, and WY–LR, generally showed positive responses within either low temperature-growth ranges or higher positive ranges. This indicates that temperature effects on TSS were strongly nonlinear and depended on the specific ES pair.

#### 3.4.2 Effects of driving factors on forest ecosystem service bundles

**Figure 7:**
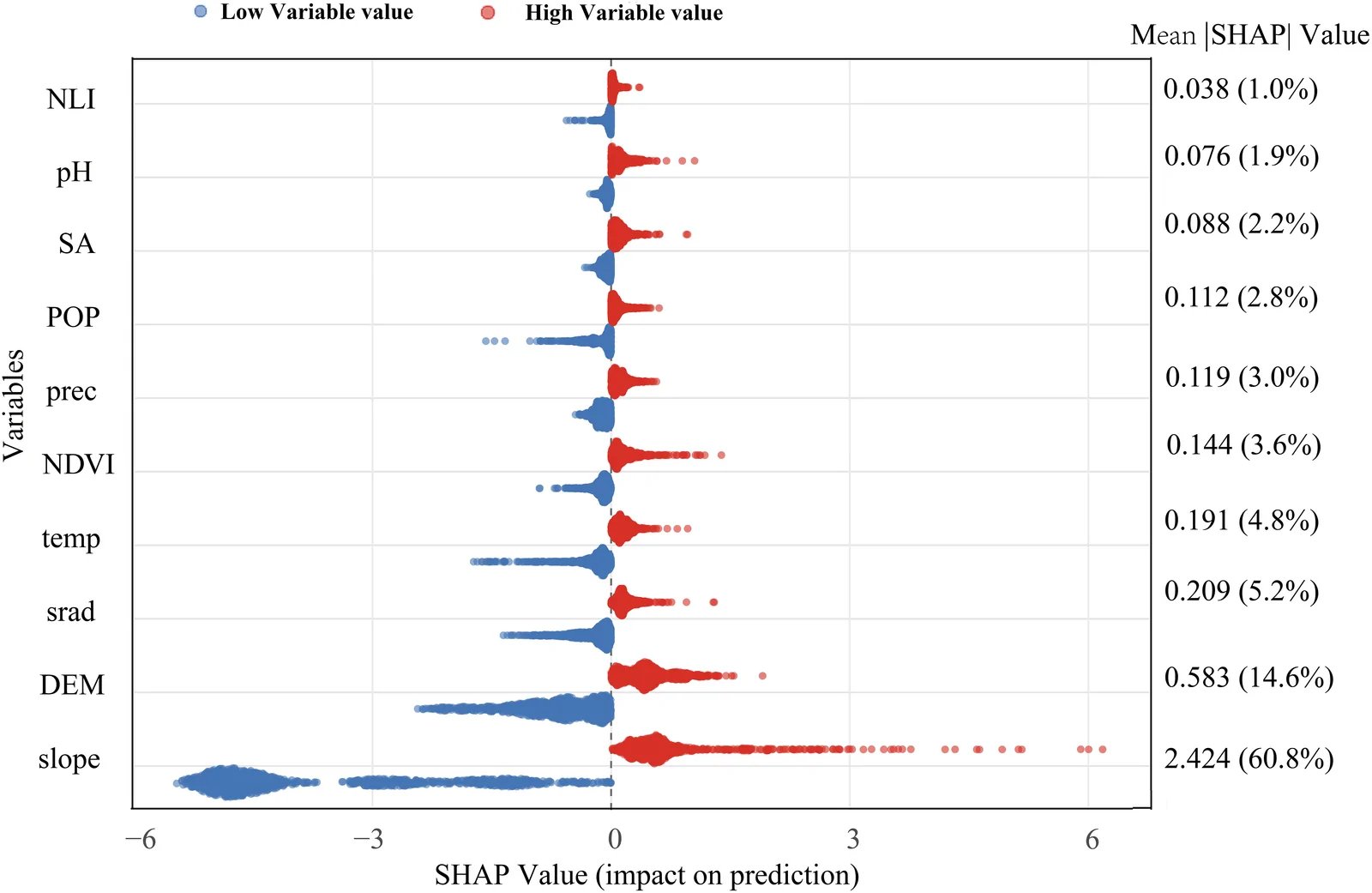
SOM feature importance map (NLI, night light data; pH, soil pH; SA, forest age; POP, population density; prec, precipitation; NDVI, normalized difference vegetation index; temp, temperature growth rate; srad, radiation growth rate; DEM, digital elevation model).

The maximum number of iterations (nrounds) was set to 3,000. Bayesian optimization indicated that the model yielded reliable results (R² = 0.74) with the following XGBoost parameters: nrounds = 2000, eta = 0.1, max_depth = 6, min_child_weight = 5, subsample = 0.8, colsample_bytree = 0.6, lambda = 2, and alpha = 1. SHAP analysis showed that (**Figure 7**), among the ten driving factors, radiation growth rate, slope, and DEM were the main factors influencing the six ES bundles (average SHAP > 0.4), with radiation growth rate having the greatest impact (average SHAP > 0.6).

**Figure 8:**
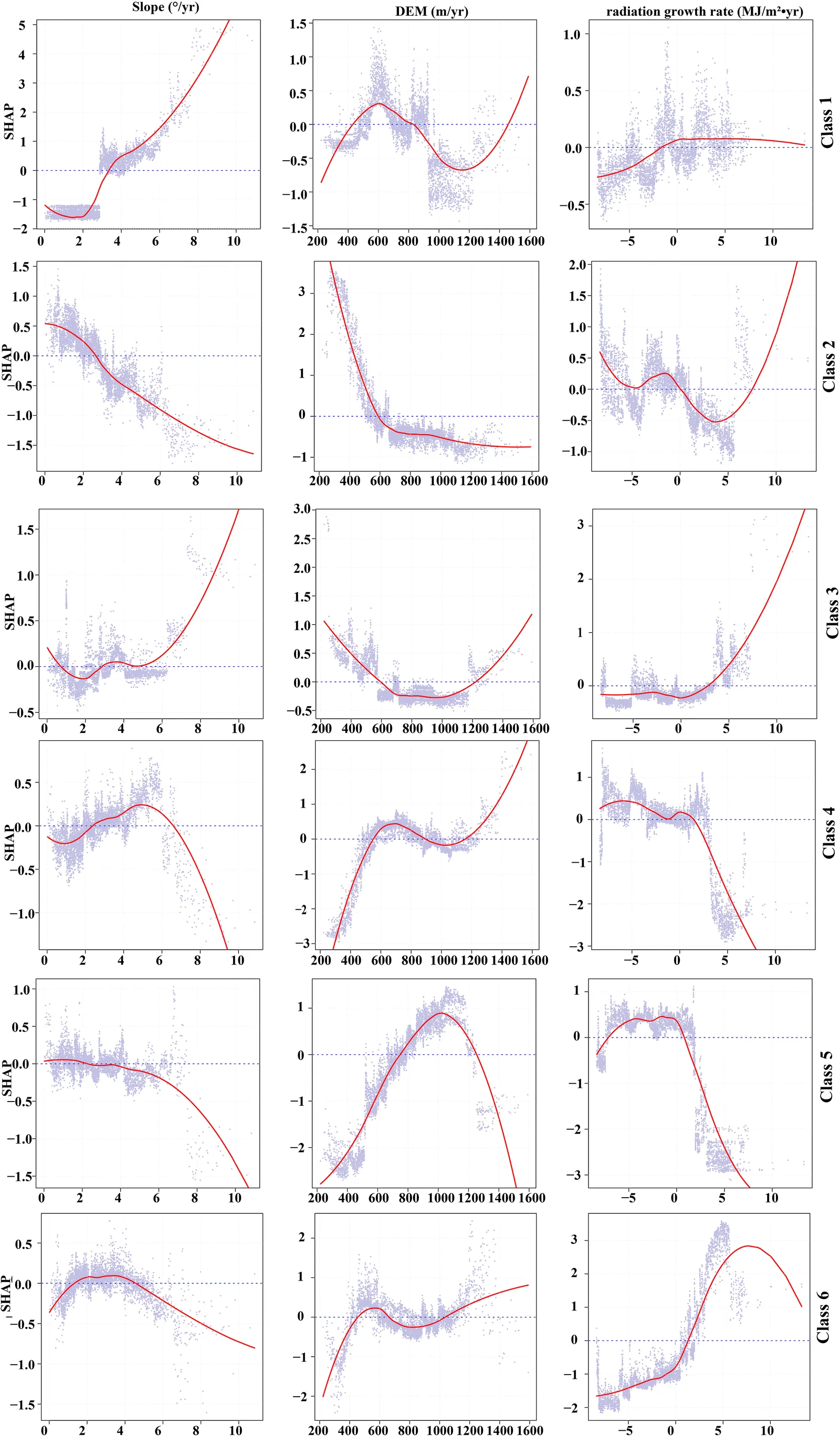
The SHAP dependence plot of six bundles (Class 1: Key synergetic bundle, Class 2: Integrated ecological bundle, Class 3: Ecological-Recreational synergy bundle, Class 4: Multi-ecological synergy bundle, Class 5: Ecological transition bundle, Class 6: Recreational development bundle).

To gain further insight into the primary factors influencing the six bundles, we selected the top three drivers based on their average SHAP values and generated SHAP dependence plots for each bundle (**Figure 8**). When the radiation growth rate exceeds -1.64 MJ·m^-2^·yr^-1^, DEM ranges between 439.38 and 837.29 m, and slope exceeds 3.36°, the key synergetic bundle is enhanced. When the radiation growth rate is less than -0.34 MJ·m^-2^·yr^-1^ or greater than 7.43 MJ·m^-2^·yr^-1^, DEM exceeds 674.15 m, and the slope is less than 2.84°, the integrated ecological bundle begins to increase.

The ecological-recreational synergy bundle exhibits a pronounced effect when the radiation growth rate exceeds 2.74 MJ·m^-2^·yr^-1^, DEM ranges between 455.20 and 1,142.28 m, and slope exceeds 2.90°. When the radiation growth rate is less than -1.54 MJ·m^-2^·yr^-1^, DEM ranges from 554.81 to 891.02 m, and slope ranges from 2.43° to 6.62°, the multi-ecological synergy bundle exhibits significant variation. When -7.70 < radiation growth rate < 0.46 MJ·m^-2^·yr^-1^, 746.32 < DEM < 1,256.62 m, and slope < 2.34°, the ecological transition bundle exhibits positive change. The synergistic effect of the recreational development bundle is maximized when the radiation growth rate exceeds 5.52 MJ·^-2^·yr^-1^, DEM ranges from 456.77 to 666.94 m, and slope ranges from 1.37° to 4.56°.

## 4 Discussion

### 4.1 Spatiotemporal dynamics of ESs and uneven effects of forest recovery

The contrasting trends among ecosystem services in the GKM suggest that forest recovery and environmental changes may generate divergent effects among ecological functions. HQ dropped markedly during 2020–2024, while LR peaked in 2015 and then declined, although it remained slightly higher in 2024 than in 1990. This pattern suggests that increases in regulation and supporting services were not uniformly accompanied by improvements in all ESs, and that enhanced vegetation functions may have coincided with reduced water yield under changing hydroclimatic conditions. This finding is consistent with previous studies showing that NPP increased markedly in the GKM from 2000 to 2019 (Zhang et al., 2023b), while WY declined and soil conservation improved. SC showed a strongly uneven spatial pattern: high values appeared only in a few isolated areas across all eight periods, while most of the region remained at relatively low levels (**Supplementary Figure 1**). The long-term mean SC was 1,437.33 t·ha^-2^, and the proportion of high-SC areas increased after 1995, especially in the northern GKM. This spatial pattern may reflect the combined influence of regional topographic and hydroclimatic conditions, because areas with relatively suitable moisture conditions and lower erosion potential are more likely to maintain higher soil conservation capacity (**Supplementary Figure 4**). The increase in SC may be partly related to long-term forest conservation policies, such as the Natural Forest Conservation Program, which may have enhanced vegetation recovery and soil stabilization capacity after its implementation. However, because the Natural Forest Conservation Program (NFCP) was mainly implemented after 1998, the increasing trends observed during the early study period (1990–2000) cannot be directly attributed to this policy alone. These changes may also reflect the combined effects of climate variability, CO_2_ fertilization effects, and natural post-disturbance forest recovery processes. After 2000, the NFCP may have further reinforced positive trends in vegetation recovery and ecosystem functioning by reducing anthropogenic disturbance and promoting forest conservation. Meanwhile, climate change may also have contributed to increases in photosynthetic activity, vegetation productivity, and carbon fixation capacity in GKM forests. The bias-corrected NPP series and recalculated CF results further suggest that these increasing trends were not mainly caused by artificial discontinuities between different NPP products. However, the marked decline in HQ during 2020–2024 and the post-2015 decline in LR further suggest that the benefits of forest recovery were not evenly transferred to habitat and cultural services. The decline in HQ after 2020 may reflect the combined effects of spatially uneven forest recovery and persistent landscape disturbance pressure. In the habitat quality assessment, HQ is influenced not only by forest cover, but also by the intensity and spatial proximity of ecological threats such as roads, human settlements, cropland, and other disturbed land-use types. Therefore, even when vegetation productivity increased, habitat quality could decline if forest recovery was spatially fragmented or if disturbance pressure increased around forest patches. The concentration of low-HQ areas in the southeastern GKM, where accessibility and human activity are relatively strong, provides a possible explanation for this spatial pattern.

The decline in LR after 2015 may reflect changes in the balance between ecological conditions, accessibility, and human-use intensity. LR is closely related to landscape attractiveness, accessibility, and recreation demand. In accessible areas, stronger human activity and recreation pressure may reduce habitat quality and landscape naturalness, thereby weakening the ecological basis of recreation services. In contrast, in ecologically sensitive areas, conservation-oriented management may limit intensive recreational development. Therefore, the decline in LR does not simply indicate reduced recreation demand, but may reflect a mismatch between recreation accessibility, ecological quality, and sustainable use.

However, the same process of vegetation recovery that improves carbon fixation, productivity, habitat conditions, and soil conservation can also reduce WY by increasing evapotranspiration (Qiu et al., 2026). As forests become denser and more productive, tree canopies intercept more rainfall, roots take up more soil water, and leaves release more water back to the atmosphere through transpiration. In simple terms, more water is used and recycled by the vegetation system, leaving less precipitation available to become surface runoff or groundwater recharge. Therefore, the decline in WY should not be understood simply as a sign of ecosystem degradation. Instead, it reflects the water cost of vegetation-driven ecological improvement. In forest systems where water is limited or unevenly distributed across seasons, this mechanism can create strong trade-offs between water supply and vegetation-related services, especially for NPP-WY, CF-WY, and SC-WY.

The spatial patterns of ESs also support this explanation. NPP and CF showed very similar distributions, with high values concentrated in the central and southern forested areas of the GKM, indicating the close link between vegetation productivity and carbon fixation. In contrast, low values of HQ and WY were mainly found in the southeastern GKM, where stronger human activity and higher population density may have affected habitat conditions and water regulation. LR values were also concentrated in the southeastern region, probably because this area has better accessibility, higher urbanization, and stronger recreation demand. CR showed lower values in the western GKM, suggesting spatial changes in local climate regulation under changing hydroclimatic conditions. Overall, these patterns show that ESs do not respond to environmental change in the same way. Therefore, understanding the interactions among ESs requires not only evaluating individual service trends, but also examining whether their temporal changes occur synchronously or competitively. Instead, climate, topography, vegetation change, and human activity interact to produce different spatial outcomes for different services.

### 4.2 TSS-based trade-off–synergy relationships and climatic thresholds

Interactions among climate, topography, vegetation, and human activities can generate diverse temporal responses among ecosystem services. These responses may lead to either coordinated changes or trade-offs depending on the environmental context and time scale (Chen et al., 2021). In the GKM, ES interactions showed clear scale-dependent characteristics. Across individual study periods, many ES pairs tended to show positive TSS values, indicating that multiple services often experienced same-direction temporal changes during short time intervals. This pattern may partly reflect the different response rates of ecosystem processes, because vegetation growth, carbon fixation, and soil-related functions may respond differently from hydrological processes under changing environmental conditions. This pattern may partly reflect a time-lag effect: vegetation-related services such as NPP, CF, and SC can respond relatively quickly to improved vegetation growth, whereas hydrological responses, especially WY, may lag behind changes in canopy structure, soil water storage, and groundwater recharge. As a result, short-term improvements in vegetation and soil-related services may temporarily coincide with stable or improved water-related services.

However, when viewed over the entire 35-year period, the cumulative water consumption associated with forest growth became more evident. As vegetation cover increased, denser canopies intercepted more precipitation, expanded root systems absorbed more soil water, and higher transpiration returned more water to the atmosphere. These processes reduced the proportion of precipitation available for runoff generation and groundwater recharge, thereby weakening WY over the long term. Therefore, the long-term ES interaction pattern reflected potential trade-offs between vegetation-related services and water supply.

The TSS results further showed that ES interactions in the GKM were mainly dominated by synergies rather than widespread trade-offs, although several ES pairs showed clear temporal shifts. Strong positive TSS relationships were mainly observed among CF–NPP, SC–CR, SC–NPP, SC–CF, SC–HQ, NPP–HQ, CR–NPP, CR–HQ, CF–CR, and CF–HQ. This indicates that forest recovery and ecological restoration generally supported the joint improvement of vegetation productivity, carbon fixation, soil conservation, habitat quality, and climate regulation. However, some ecosystem service pairs exhibited weakened synergies or temporary trade-offs during specific periods, particularly between vegetation-related services and water-related services during 2010–2015. These shifts suggest that the positive effects of vegetation recovery were not evenly shared across all services during this period. Because TSS is calculated from the direction and magnitude of temporal changes in paired ESs, negative TSS values become larger in magnitude when one group of services continues to improve while another remains stable or declines. During 2010–2015, vegetation-related services such as NPP, CF, and SC continued to benefit from forest recovery and vegetation growth, whereas WY, HQ, and some CR-related relationships did not improve synchronously. The negative TSS values observed in some WY–vegetation relationships may be related to the increased water demand of more productive forests, which can reduce runoff generation and groundwater recharge through higher canopy interception, root water uptake, and transpiration. In addition to vegetation water demand, permafrost degradation may represent an additional potential mechanism contributing to WY-related variability. The GKM is an important permafrost-affected region in Northeast China, and climate warming has led to rapid permafrost degradation in this region. Shan et al. (2023) reported that the permafrost area in Northeast China decreased from 3.31 × 10^5^ km² to 2.70 × 10^5^ km² during 2000–2020, and that water released from degrading permafrost can strongly affect river runoff in high-altitude source regions such as the Greater and Lesser Khingan Mountains. The active-layer freeze–thaw process can also alter soil hydrothermal dynamics in the Great Hing’an Mountains, including soil temperature and moisture conditions (Dong et al., 2021). These changes may affect groundwater storage, hydraulic connectivity, surface runoff generation, and root-zone soil moisture, thereby influencing both WY and vegetation water uptake. Recent studies further showed that soil freeze–thaw changes and permafrost degradation can influence vegetation growth and NPP in Northeast China (Hong et al., 2025). Therefore, the strong fluctuations in WY and its trade-offs with productivity-related services may partly reflect the combined effects of vegetation recovery and permafrost-related hydrological changes. However, because long-term and spatially explicit permafrost datasets consistent with the spatial and temporal resolution of this study were not available, permafrost degradation was not included as an explicit driving factor. Future studies should further integrate permafrost extent, active-layer thickness, ground ice content, and groundwater dynamics to better quantify freeze–thaw effects on ES interactions in the GKM. Meanwhile, the trade-offs involving CR and HQ suggest that increases in productivity and carbon fixation did not necessarily translate into better local climate regulation or habitat quality. This may be associated with spatially uneven forest recovery, climate stress, changes in forest structure, or local human disturbance. The increased synergistic relationships during 2010–2015 may reflect a period when vegetation recovery, ecosystem restoration, and favorable environmental conditions jointly promoted more coordinated changes among multiple ecosystem services. The concentration of synergistic relationships in the northern and central forest regions may be associated with relatively intact forest conditions, continuous vegetation cover, and lower disturbance intensity in these areas. These ecological characteristics may enhance the consistency of temporal responses among ecosystem services and promote coordinated changes across multiple services. Therefore, although ES interactions were generally synergy-dominated, the 2010–2015 period highlights the potential instability of these synergies under changing climate and management conditions. Previous studies have shown that regions with annual precipitation of 400–800 mm are particularly sensitive to climate change (Yang et al., 2022). Our precipitation trend analysis further suggests that very high precipitation levels above 800 mm may weaken synergistic relationships in the GKM (**Supplementary Figure 5**).

The driver analysis further showed that climatic variables were dominant drivers of temporal changes in TSS, with precipitation growth rate, radiation growth rate, and temperature growth rate jointly accounting for 52.1% of the mean absolute SHAP contribution. Among the ten explanatory variables, precipitation growth rate was the most influential factor, accounting for 21.7% of the mean absolute SHAP contribution, followed by radiation growth rate (18.1%), elevation (13.7%), and temperature growth rate (12.3%). These results indicate that hydroclimatic variability and topographic conditions jointly regulated the spatial variation of ES trade-off–synergy patterns. This result indicates that changes in ES trade-offs and synergies in the GKM were mainly shaped by hydroclimatic variability rather than by a single environmental factor. The SHAP results further revealed that the effects of climate drivers on TSS were nonlinear, with specific climatic-change ranges associated with variation in model-estimated TSS responses. SHAP dependence analysis revealed nonlinear response patterns of ecosystem service interactions to climatic variables (**Supplementary Figure 3**). Across different ecosystem service pairs, positive SHAP responses were generally associated with precipitation growth rates ranging approximately from −0.74 to 0.03 mm·yr^-1^, radiation growth rates from −8.00 to 5.59 MJ·m^-2^·yr^-1^, and temperature growth rates from −3.22 to 1.74 °C·yr^-1^ under the climatic-change conditions examined in the GKM. These results suggest that ES trade-off–synergy patterns were not controlled by monotonic climatic effects, but instead responded to variable-specific climatic ranges. These model-derived response ranges suggest that ES interactions respond to broad but nonlinear climatic conditions, with different ecosystem service pairs exhibiting distinct response intervals. However, these ranges represent statistical associations identified by the XGBoost-SHAP framework rather than causal ecological thresholds, and they should not be directly interpreted as universal climatic limits or extrapolated to other boreal forest regions without further validation.

These findings suggest that climate change does not affect ES interactions in a simple linear way. Climatic conditions within these response ranges were associated with higher positive SHAP contributions, whereas conditions outside these ranges showed weaker or negative contributions to the modeled TSS responses (Xu et al., 2024). Therefore, ecological restoration in the GKM should not only aim to increase vegetation cover, but should also consider whether local climatic and hydrological conditions can support the water demand of recovering forests (Persson et al., 2024; Singh et al., 2024; Susaeta et al., 2023). Under climatic conditions that constrain ecosystem water availability, vegetation recovery may increase competition for limited water resources and potentially strengthen trade-offs between water supply and other ecological services (Moser, 2024; L. Xu et al., 2025b; Yang et al., 2023). Future ecological restoration and conservation projects in the GKM should therefore place greater emphasis on coordinated management of vegetation growth, water availability, and climate adaptation.

### 4.3 Common boreal forest patterns and regional specificity of the GKM

To clarify the regional specificity of our findings, we compared our results with previous studies from adjacent forest regions in Northeast Asia and other boreal forests. Some patterns observed in the GKM are consistent with broader characteristics of the Northeast China forest belt. For example, Niu et al. (2021) showed that the Greater Khingan Mountains, Lesser Khingan Mountains, and Changbai Mountains jointly form the main forest belt in Northeast China, where large-scale forest cover supports high levels of water retention, soil conservation, biodiversity, and carbon-related services. They also reported widespread synergies between water retention and soil conservation, and between water retention and biodiversity, especially in forested mountain areas such as the Lesser Khingan Mountains and Changbai Mountains. Similarly, Zhu et al. (2024) emphasized that ecosystem service interactions and bundles in China’s Northeast Forest Belt are spatially scale-dependent and should be considered in spatially targeted ecosystem management. These studies support our finding that forest-dominated regions in Northeast China commonly show close linkages among regulating and supporting services.

However, several patterns in the GKM also differed from those reported in adjacent mountain regions. In the Changbai Mountain Region, Liu et al. (2026) found that soil retention and carbon storage increased from 1990 to 2020, whereas water yield and habitat quality decreased, and the dominant ES interactions included trade-offs between water yield and soil retention and synergies between water yield and habitat quality. In contrast, our study found that synergies among NPP, CF, SC, HQ, and CR were dominant in the GKM, whereas WY-related relationships were more likely to show trade-offs. This difference suggests that although carbon- and soil-related service improvements are common across Northeast China’s forested mountains, the hydro-ecological interactions in the GKM are more strongly constrained by cold-temperate climate conditions, hydrothermal variability, and regional forest recovery processes.

Comparisons with Siberian and other boreal forests further highlight both common boreal characteristics and regional differences. Russian boreal forests provide important carbon-related ecosystem functions but are increasingly affected by climate change; radiation increases, longer growing seasons, and elevated CO_2_ can enhance productivity, whereas heat waves, drought, fires, and insect outbreaks may weaken carbon sinks and forest stability (Schaphoff et al., 2016). In broader boreal forest systems, trade-offs between timber production, carbon storage, biodiversity, deadwood, and other non-provisioning services have also been widely reported (Mazziotta et al., 2022). Compared with these Siberian and boreal forest systems, where timber extraction, fire, permafrost processes, and climate-driven disturbances often strongly shape ES trade-offs, the GKM shows a more distinct pattern related to ecological protection, logging restrictions, vegetation recovery, and climate-driven water constraints. Therefore, the GKM shares general boreal forest features, such as strong climate sensitivity and carbon-related synergies, but its TSS pattern also reflects a region-specific pathway in which vegetation recovery enhances productivity- and carbon-related services while water-related trade-offs remain pronounced.

### 4.4 Implications and suggestions for ecological management

The ecosystem service bundle results revealed clear spatial differences in ES combinations and management priorities across the GKM. These bundles provide a basis for differentiated spatial management by identifying areas with distinct ecological functions, dominant ES relationships, and conservation needs. By integrating ES bundle distributions, TSS relationships, and SHAP-derived response ranges, this study proposed regional-scale management suggestions for the GKM. These suggestions aim to identify priority areas, potential conflict zones, and possible management directions rather than serve as direct site-specific prescriptions. Specific actions, including species selection, restoration intensity, tourism development, and strict protection measures, should be further evaluated using field surveys, forest inventory data, local management plans, and socioeconomic assessments.

Among the six ES bundles, the multi-ecological synergy bundle may represent a priority area for conservation attention and long-term monitoring. This bundle accounted for the largest proportion of the study area and was mainly distributed in the central GKM, indicating its important role in maintaining regional ecological stability. SHAP dependence analysis showed that this bundle was associated with radiation growth rates lower than −1.54 MJ·m^-2^·yr^-1^, DEM values ranging from 554.81 to 891.02 m, and slopes ranging from 2.43° to 6.62°. These results suggest that moderate-elevation and gentle-slope areas with relatively weak radiation increases were associated with the maintenance of multiple ecological synergies. Therefore, management in this bundle should prioritize ecological stability, biodiversity conservation, and water resource regulation while avoiding intensive land-use disturbance. However, the appropriate protection level and restoration intensity should be determined through field-based ecological assessments and local management planning.

The integrated ecological bundle, mainly distributed in the northern and western GKM, also represents an area with high ecological conservation value. In this bundle, HQ, WY, and CR showed synergistic relationships, indicating that habitat quality, water yield, and climate regulation were jointly associated with favorable environmental conditions. Therefore, ecosystem conservation should be strengthened in this region, with restoration measures focusing on improving forest structure and maintaining soil and water conservation functions. Studies in northeastern China have shown that mixed forests generally exhibit better ecological stability and multifunctional performance (Schnabel et al., 2024). Thus, future restoration in this bundle could consider optimizing forest structure and maintaining native species diversity. However, specific species selection, including the use of cold- or drought-tolerant native species, should be determined according to local site conditions, field surveys, and forest management objectives (McKinney et al., 2023; Zhao et al., 2022). Restoration practices should also avoid excessive afforestation or overly dense forest stands, as these may increase evapotranspiration and further reduce WY.

The ecological-recreational synergy bundle exhibited strong synergies between ecological and recreational functions and may require conservation-oriented management. Management in this bundle should emphasize maintaining both ecological integrity and recreational benefits. Water resource regulation, soil conservation, and habitat protection could be prioritized to reduce the potential impacts of recreational activities on ecological quality. For the recreational development bundle, recreation-oriented use may be considered in areas with suitable ecological conditions and accessibility. However, improvements in tourism infrastructure should be based on carrying-capacity assessments, ecological impact evaluations, and local planning constraints. Ecological protection should therefore be integrated into tourism planning to avoid potential declines in habitat quality, reduced water regulation capacity, and increased landscape fragmentation.

The key synergetic bundle, although covering only a small proportion of the study area, represents a priority area for targeted management of important ecological functions. Management in this bundle should focus on soil restoration, erosion control, and the maintenance of local climate regulation functions. Due to its limited spatial extent, targeted and fine-scale management approaches may be more appropriate than broad restoration measures. The ecological transition bundle requires more balanced management because improvements in one service may be accompanied by declines in another. Vegetation restoration in this bundle should be coordinated with soil and water conservation measures. Management should avoid excessive vegetation expansion that may increase evapotranspiration and potentially reduce water yield, while maintaining soil stability and carbon fixation capacity.

The TSS framework provides additional ecological and management information beyond individual ES maps and ES bundle classifications. By incorporating both the direction and strength of pairwise ES interactions, TSS helps reveal the underlying interaction structure among ecosystem services and provides a spatially explicit measure of multifunctionality dynamics. Conventional correlation-based approaches can identify the overall statistical direction of ES relationships, but they provide limited information on the spatial distribution and strength of trade-offs and synergies at the pixel scale. In contrast, TSS describes where specific interactions occur and how strongly they are expressed across the landscape. The moderate agreement between TSS and partial correlation analyses supports the consistency of TSS-derived relationship directions, while the incomplete agreement suggests that TSS captures spatially heterogeneous interaction patterns that are not fully represented by global statistical correlations. This distinction is important because areas with similar overall ES levels may experience different management challenges. In one area, high ES levels may be associated with strong synergies among services, whereas in another area, similar ES levels may coexist with strong trade-offs between specific service pairs. In this study, the dominant synergies among NPP, CF, SC, HQ, and CR were associated with concurrent improvements in multiple ecological functions, whereas persistent trade-offs involving LR and variability in WY-related pairs indicate potential constraints for future forest management.

The shift toward stronger trade-offs during 2010–2015 suggests that increases in vegetation-related services may not always correspond to balanced changes among ecosystem functions, particularly under changing hydroclimatic conditions. By linking TSS with XGBoost–SHAP analysis, we further identified climatic conditions associated with variations in synergistic responses. These results suggest that ecological restoration in the GKM should not simply aim to increase vegetation cover, but should also consider local water availability, radiation changes, and temperature variations. From a management perspective, areas with strong positive TSS values represent locations where multiple ecosystem services exhibited consistent same-direction temporal changes with relatively large composite change magnitude. These areas can provide useful information for identifying zones with coordinated ecosystem service trajectories, particularly where carbon fixation, productivity, soil conservation, habitat quality, and climate regulation changed synchronously. In contrast, areas with strong negative TSS values indicate locations where paired ecosystem services showed opposite-direction temporal changes, suggesting potential management conflicts where the improvement of one service may be accompanied by the decline or slower recovery of another. Therefore, TSS-based identification of coordinated and conflicting zones can complement traditional ecosystem service assessments by revealing the temporal interaction structure among services. Areas with strong negative TSS values can be considered potential conflict zones, where paired ecosystem services exhibited opposite temporal trajectories and may require balanced management strategies. In these areas, management should avoid single-objective restoration and instead adopt more balanced strategies, such as regulating vegetation intensity, protecting water resources, reducing excessive recreational pressure, and maintaining habitat connectivity. Therefore, TSS provides a practical basis for moving from general bundle-based zoning to more targeted management of specific ES conflicts and synergies.

However, TSS should be interpreted as a diagnostic rather than causal metric. It identifies the signed strength of ES co-changes, but it does not by itself prove the ecological mechanisms causing these relationships. Its reliability depends on the accuracy, normalization, and temporal consistency of ES estimates. Therefore, TSS should be combined with driver analysis, field knowledge, and process-based evidence when used to inform ecological management.

The differences in model explanatory power among service pairs also provide useful information about the complexity of forest ES interactions. Although most TSS models showed acceptable predictive performance, several service pairs, especially NPP–HQ and other HQ-related relationships, were less well explained by the selected predictors. This issue is particularly relevant for the NPP–HQ relationship. Because this study was restricted to forest pixels, the weak model performance cannot simply be attributed to broad land-use differences. Instead, it more likely reflects the fact that NPP and HQ represent different dimensions of forest ecosystem functioning. NPP mainly describes vegetation productivity and is closely associated with hydrothermal conditions, radiation availability, photosynthetic activity, and stand growth. By contrast, HQ represents habitat suitability and ecological integrity within forest landscapes, and may be more sensitive to forest structural complexity, canopy condition, stand age heterogeneity, habitat continuity, local degradation, and disturbance history than to broad climatic gradients alone.

The weak explanatory power of the NPP–HQ model therefore suggests that productivity–habitat relationships within forests may be shaped by finer-scale and history-dependent processes. A forest area with high productivity does not necessarily have high habitat quality if it has a simplified stand structure, low habitat continuity, strong edge effects, or a legacy of previous disturbance. Conversely, forests with moderate productivity may still maintain high habitat quality when they have greater structural complexity, better canopy continuity, lower disturbance intensity, and more stable habitat conditions. In this study, several potentially important variables, including stand density, canopy structure, understory condition, deadwood availability, species composition, previous disturbance history, fire and pest disturbance, and detailed forest management records, were not fully represented by the ten raster-based predictors. Although external threat proximity and forest-edge effects may also influence HQ, these factors were not the main focus because the analysis was limited to forest pixels. These unmeasured forest-internal processes and possible spatial scale mismatches may have weakened the ability of XGBoost to explain the spatial variation in NPP–HQ TSS.

Accordingly, SHAP-derived drivers and thresholds should be interpreted in relation to model performance. For service pairs with moderate to high test-set R² values, SHAP results provide useful evidence for identifying dominant drivers and potential response thresholds. However, for low-performance models, especially NPP–HQ and other HQ-related pairs, SHAP patterns should be regarded as exploratory rather than definitive. In this study, the general conclusions about climatic controls on TSS were therefore based mainly on patterns that were consistent across multiple service pairs and were supported by models with acceptable predictive performance, rather than on any single low-R² relationship. This distinction is important for avoiding overinterpretation of machine-learning explanations and for recognizing that some forest ES interactions may require additional information on forest structure, disturbance history, management legacy, and habitat continuity to be better explained.

Several limitations should be acknowledged. First, because of data availability, the selected drivers were mainly limited to commonly available raster-based environmental variables. Future studies should incorporate more detailed mechanism-related variables, such as forest structure, species composition, soil properties, disturbance history, and human activity intensity, to improve the ecological interpretation of ES interactions. Second, although TSS can identify the signed strength of ES co-changes, it cannot directly reveal causal mechanisms. Future research could combine TSS with process-based models, field observations, or causal inference methods (Zhang and Wang, 2025) to better explain how different drivers shape ES trade-offs and synergies. Finally, multi-scale analyses are needed to test whether TSS-based interaction patterns remain stable across different spatial resolutions, temporal intervals, and forest management units. Second, the use of a long-term forest mask ensured that all analyses focused on persistent forest ecosystems and minimized the confounding effects of land-cover conversion among different ecosystem types. However, this approach also excluded areas experiencing deforestation, afforestation, urban expansion, or other land-cover transitions. Therefore, the detected ES dynamics mainly represent within-forest changes rather than landscape-scale changes associated with LUCC. This limitation is particularly relevant for HQ, because habitat loss, fragmentation, and forest–non-forest boundary effects may strongly influence habitat conditions but cannot be fully captured within persistent forest areas. Even within the forest mask, HQ may still be affected by forest degradation, structural simplification, disturbance history, and changes in habitat continuity. Future studies integrating dynamic land-cover trajectories and landscape configuration metrics could provide a more comprehensive understanding of ES changes across the entire landscape.

## 5 Conclusions

This study developed a TSS index to quantify both the direction and intensity of ES interactions in the GKM from 1990 to 2024. By combining ES assessments, TSS, SOM, and XGBoost–SHAP analyses, we established a framework to characterize ES interaction patterns, identify dominant environmental drivers, and support adaptive management. The main conclusions are as follows: (1) The seven ESs in the GKM exhibited significant spatiotemporal variations during 1990–2024. SC, CF, NPP, and CR showed overall increasing trends, whereas WY declined. HQ decreased markedly during 2020–2024, while LR peaked in 2015 and then declined, indicating that improvements in ESs were not consistently maintained across all services. (2) TSS analysis showed that synergistic relationships were mainly observed among CF–NPP, SC–CR, SC–NPP, SC–CF, SC–HQ, NPP–HQ, CR–NPP, CR–HQ, CF–CR, and CF–HQ, whereas LR-related pairs exhibited persistent trade-offs and WY-related pairs showed greater spatial and temporal variability. (3) Climatic drivers showed nonlinear associations with TSS variation and exhibited distinct model-derived response ranges, indicating that ES interactions responded to variable-specific climatic changes rather than simple linear relationships. (4) SOM analysis identified six ES bundles across the GKM, revealing clear spatial differences in ecosystem service combinations and management requirements. (5) Overall, the TSS framework provides a useful spatial diagnostic tool for characterizing ES trade-offs and synergies by jointly representing interaction direction and composite magnitude of ES co-change rather than causal interaction strength. It helps identify areas with strong, contrasting, or temporally variable ES interaction patterns and provides spatial information for management prioritization. However, TSS should not be interpreted as a direct causal measure and should be combined with environmental driver analysis, field knowledge, and process-based evidence when informing management decisions.

## Data Availability

The processed datasets are not publicly deposited but are available from the corresponding author upon reasonable request. Use of the original datasets is subject to the terms and conditions of the respective data providers. Requests to access the datasets should be directed to LD, farrell0503@126.com.
