# Supplementary material for: Multi-decadal dynamics of ecosystem service interactions in boreal forests (NE China): trade-off synergy strength, drivers, and thresholds
Source: Front Plant Sci. 2026 Aug 24;17:1921235. doi: 10.3389/fpls.2026.1921235 (PMC13547479; doi:10.3389/fpls.2026.1921235)
Supplement: Supplementary file 1 [file DataSheet1.pdf]

Supplementary Material

Multi-decadal dynamics of ecosystem service interactions in boreal forests (NE China): Trade-off synergy strength, drivers, and thresholds

Xueqing Wu[1,2], Wei Lu[3], Lingbo Dong[1,2]*

[1] College of Forestry, Northeast Forestry University, Harbin 150040, China

[2]Key Laboratory of Sustainable Forest Ecosystem Management, Ministry of Education, School of Forestry, Northeast Forestry University, Harbin 150040, China

[3]College of Forestry, Hebei Agricultural University, Baoding, Hebei 071000, China

***Correspondence:**
Corresponding Author
farrell0503@126.com

Catalog

1 ES evaluation Methods 3

1.1 Water yield 3

1.2 Soil conservation 3

1.3 Habitat quality 3

1.4 Carbon fixation and oxygen release 4

1.5 Climate regulation 4

1.6 Leisure and recreation 4

1.7 Net primary productivity 5

2 Sensitivity assessment of the leisure and recreation index 5

3 Robustness assessment of TSS framework 6

4 Supplementary Figures 13

References 18

Table captions

Table S1. Leave-one-component-out sensitivity analysis of the leisure and recreation index

Table S2. Comparison between TSS-derived interaction directions and correlation-based classifications.

Table S3. Period-level agreement between TSS and correlation-based classifications.

Table S4. Sensitivity of TSS–ASI consistency under different absolute TSS thresholds.

Table S5. Sensitivity of dominant interaction patterns after excluding the CF–NPP pair.

Table S6. Bootstrap and leave-one-pair-out sensitivity analyses of TSS-derived interaction patterns

Table S7. Detailed parameters and cross-validation performance of XGBoost models for predicting long-term TSS patterns.

Figure captions

Figure S1. Spatial patterns of the forest ecosystem services in Great Khingan Mountains between 1990 and 2024.

Figure S2. Spatial patterns of the trade-off synergy strength in Great Khingan Mountains in 7 periods.

Figure S3. SHAP dependence plots showing the response patterns of environmental drivers for different TSS relationships.

Figure S4. Slope of meteorological factor variation.

Figure S5. The precipitation variation curve of Great Khingan Mountains from 1990 to 2024.

1 ES evaluation Methods

1.1 Water yield

WY was used to represent the water supply capacity of forest ecosystems. Annual water yield was calculated using the Water Yield module of the InVEST model. This module combines annual precipitation with the Budyko water-heat coupling balance assumption to estimate annual water yield (Wang et al., 2022):

$$Y(x) = \left(1 - \frac{AET(x)}{P(x)}\right) \cdot P(x) \tag{1}$$

where *AET(x)* is the actual evapotranspiration of pixel x; *P(x)* is the annual precipitation of pixel x. Root depth and plant-available water content were derived from the HWSD. The Zhang coefficient was calibrated iteratively by comparing simulated water yield with records from the Water Resources Bulletin, and was ultimately set to 1.5.

1.2 Soil conservation

SC was used to represent the capacity of forest ecosystems to reduce soil erosion and retain soil. SC was estimated using the InVEST sediment delivery ratio model. This model incorporates the plot's inherent capacity to retain upstream sediments, as determined by the Universal Soil Loss Equation (USLE) (Shifaw et al., 2025):

$$SC = RKLS - USLE \tag{2}$$

$$RKLS = R \times K \times LS \tag{3}$$

$$USLE = R \times K \times LS \times P \times C \tag{4}$$

where SC is soil conservation (t); RKLS is the potential soil erosion for each grid under current land use without C and P factor adjustments, representing the soil erosion rate for bare land (i.e., potential soil erosion); USLE represents the potential soil erosion per grid cell under current land use with C and P factor adjustments (i.e., actual soil erosion); R is the rainfall erosivity factor ($MJ \cdot mm \cdot hm^{-2} \cdot h^{-1} \cdot a^{-1}$); K is the soil erodibility factor; LS is the slope length and steepness factor; C is the cover and management factor; and P is the support practice factor.

1.3 Habitat quality

HQ was used to represent the supporting capacity of forest ecosystems for biodiversity conservation. The InVEST Habitat Quality module was used to generate habitat quality maps at the pixel level (Lopezosa et al., 2023; Wu et al., 2021). Following the InVEST user guide and considering the geographical environment of the GKM, this study selected construction land, cultivated land, bare land, nighttime light index, railways, highways, and GDP as sources of ecological threats. The maximum stress distance and weight for each threat source were assigned based on relevant case studies, as were the habitat suitability and sensitivity to stress factors for different habitat types:

$$Q_{xj} = H_j \left(1 - \left(\frac{D_{xj}^z}{D_{xj}^z - K^z}\right)\right) \tag{5}$$

where Q_{xj} is the habitat quality of grid cell x in land cover type j; H_j represents the habitat suitability of land cover type j; D_{xj} is the stress level (i.e., degradation risk index) experienced by grid cell x in land cover type *j*; z is the model's default parameter, typically set to 2.5, and K is the semi-saturation constant, equivalent to half the maximum habitat degradation value. K was set to 0.5, and the model was run once for calibration. The highest habitat degradation value was then observed before the configuration was finalized.

1.4 Carbon fixation and oxygen release

CF was used to represent the gas regulation capacity of forest ecosystems. It reflects the ability of vegetation to fix carbon dioxide and release oxygen through photosynthesis. In this study, CF was calculated based on NPP data following the Technical Guidelines for Gross Ecosystem Product (GEP) Accounting (Shang, 2024):

$$Q_{op} = NPP_i \times S_i \times (1.62 + 1.2) \tag{6}$$

where Q_{op} is the gas regulation amount (t) for the evaluation unit; NPP_i represents the NPP value (kg $C \cdot m^{-2}$) for each evaluation unit; S_i is the area of the evaluation unit (km^2). All raster data used in this study have a uniform grid resolution of 1 km × 1 km; therefore, S_i is equivalent to 1 km^2.

1.5 Climate regulation

Climate regulation (CR) was used to represent the capacity of forest ecosystems to regulate regional heat and moisture conditions. This study used the annual heat-moisture index (AHM) to characterize the regional heat-moisture condition (Li et al., 2025). A higher AHM value indicates drier conditions, while a lower value indicates more humid conditions. Because the direction of AHM is opposite to climate regulation capacity, AHM was inversely normalized so that higher CR values represented stronger climate regulation capacity.

$$AHM = \frac{MAT + 10}{\frac{MAP}{1000}} \tag{7}$$

where MAT is the mean annual temperature (°C); MAP is the mean annual precipitation (mm).

The inverse normalization was calculated as follows:

$$CR_i = \frac{AHM_{max} - AHM_i}{AHM_{max} - AHM_{min}} \tag{8}$$

1.6 Leisure and recreation

Leisure and recreation (LR) was used to represent the cultural service capacity of forest ecosystems. It reflects the non-material benefits that people obtain from natural and semi-natural landscapes,

which are influenced by both landscape quality and accessibility. The recreational index (RECRI) was developed in this study to evaluate LR services in the GKM, drawing on the framework of Paracchini et al. (2014):

$$RECRI = Si\sum\left(Road + POP + GDP + NDVI + TRI\right) \tag{9}$$

where S_i is the ecosystem area (km²); Road is the road proximity score; POP is the population density score; GDP represents the purchasing power score; NDVI represents the scenic appeal score; and TRI represents the topographic relief index score. This study was conducted using a 1 km grid; therefore, S_i represents 1 km². Road proximity scores were calculated using Euclidean distance analysis in ArcGIS, with the following values assigned: 7 points for distances < 3,000 m, 2 points for 3,000–5,000 m, and 0 points for distances > 5,000 m. Population density and purchasing power scores were derived using focal statistics analysis. Population density was analyzed within a 10 km radius, while purchasing power was analyzed within a 5 km radius. Values were assigned using the natural breaks (Jenks) method, increasing sequentially from lowest to highest. Topographic relief was derived from terrain undulation, with values assigned in reverse order using the natural breaks (Jenks) method. Scenic appeal was categorized into five classes based on NDVI, with scores assigned from 0 to 10 in ascending order of visual quality.

1.7 Net primary productivity

Net primary productivity (NPP) was used to represent the supporting capacity of forest ecosystems related to vegetation productivity. It reflects plant growth, vegetation condition, and the ecological basis for carbon accumulation and other regulating services (Li et al., 2016).

2 Sensitivity assessment of the leisure and recreation index

To evaluate the sensitivity of the leisure and recreation index to its individual components, a leave-one-component-out analysis was conducted. Because the RECRI framework does not apply subjective weights, sensitivity was assessed by separately excluding the GDP-based purchasing-power score, population-density score, and road-proximity score from the full index. An additional ecological-component-only scenario, retaining only the NDVI-based scenic appeal score and topographic relief score, was included as an extreme comparison rather than a strict leave-one-component-out scenario. All scenarios were evaluated using a common spatial mask comprising 17,478 grid cells with valid data across all assessment years. Sensitivity was assessed using Pearson and Spearman spatial correlations with the full RECRI, consistency in the regional peak year, pixel-level Sen-slope correlations, and agreement in long-term trend direction.

The leave-one-component-out analysis showed that the spatial pattern of LR remained highly stable after excluding road proximity or population density, with mean Pearson correlations of 0.971 and 0.939, respectively. Excluding the GDP-based purchasing-power component resulted in a lower but still positive spatial correlation with the full RECRI, with a mean Pearson correlation of 0.784. The regional LR peak remained in 2015 after excluding population density or road proximity, whereas it shifted to 2005 after excluding GDP. Trend-direction agreement with the full RECRI was 100.0% for the road-exclusion scenario, 73.4% for the population-exclusion scenario, and 69.6% for the GDP-exclusion scenario. These results indicate that the broad spatial pattern of LR was generally stable, whereas the timing and magnitude of the 2015 regional maximum were sensitive to the GDP-based purchasing-power component.

Table S 1 Leave-one-component-out sensitivity analysis of the leisure and recreation index

Scenario	Mean Pearson r	Mean Spearman ρ	Trend-direction agreement (%)	Sen-slope Pearson r	Sen-slope Spearman ρ	Peak year
Remove GDP	0.784	0.821	69.6	0.606	0.633	2005
Remove POP	0.939	0.942	73.4	0.913	0.810	2015
Remove Road	0.971	0.948	100.0	1.000	1.000	2015
Ecological components only	0.198	0.263	6.2	0.164	0.205	2010

3 Robustness assessment of TSS framework

To evaluate the robustness of the TSS framework, we conducted multiple sensitivity analyses. First, the consistency between TSS-derived interaction classifications and ASI-based dominant patterns was assessed under different absolute TSS thresholds (|TSS| > 0, 0.05, 0.10, 0.15, and 0.20). Second, because CF was calculated using NPP as the fundamental input, the potential influence of this inherent dependency was examined by removing the CF–NPP pair. Third, bootstrap resampling and leave-one-pair-out analyses were performed to evaluate whether dominant interaction patterns remained stable under perturbations of sampling and ecosystem service combinations. Correlation-based approaches (Pearson, Spearman, and partial correlation) were additionally provided as complementary comparisons rather than independent validation criteria.

Table S 2. Comparison between TSS-derived interaction directions and correlation-based classifications.

Period	Pair	TSS_type	Pearson_type	Spearman_type	Partial_type
1990_1995	WY_NPP	Synergy	Trade-off	Trade-off	Synergy
1990_1995	WY_SC	Synergy	Synergy	Synergy	Synergy
1990_1995	WY_HQ	Synergy	Synergy	Synergy	Synergy
1990_1995	WY_CF	Synergy	Trade-off	Trade-off	Trade-off
1990_1995	WY_CR	Synergy	Synergy	Synergy	Synergy
1990_1995	WY_LR	Synergy	Trade-off	Trade-off	Trade-off
1990_1995	NPP_SC	Synergy	Synergy	Synergy	Trade-off
1990_1995	NPP_HQ	Synergy	Trade-off	Trade-off	Synergy
1990_1995	NPP_CF	Synergy	Synergy	Synergy	Synergy
1990_1995	NPP_CR	Synergy	Trade-off	Trade-off	Synergy
1990_1995	NPP_LR	Synergy	Synergy	Trade-off	Synergy
1990_1995	SC_HQ	Synergy	Synergy	Synergy	Synergy
1990_1995	SC_CF	Synergy	Synergy	Synergy	Synergy
1990_1995	SC_CR	Synergy	Synergy	Synergy	Trade-off
1990_1995	SC_LR	Synergy	Synergy	Trade-off	Trade-off
1990_1995	HQ_CF	Synergy	Trade-off	Trade-off	Trade-off

Period	Pair	TSS_type	Pearson_type	Spearman_type	Partial_type
1990_1995	HQ_CR	Synergy	Synergy	Synergy	Synergy
1990_1995	HQ_LR	Synergy	Synergy	Synergy	Synergy
1990_1995	CF_CR	Synergy	Trade-off	Trade-off	Trade-off
1990_1995	CF_LR	Synergy	Synergy	Trade-off	Trade-off
1990_1995	CR_LR	Synergy	Trade-off	Synergy	Synergy
1995_2000	WY_NPP	Synergy	Trade-off	Trade-off	Trade-off
1995_2000	WY_SC	Synergy	Trade-off	Trade-off	Trade-off
1995_2000	WY_HQ	Trade-off	Synergy	Synergy	Trade-off
1995_2000	WY_CF	Synergy	Trade-off	Trade-off	Synergy
1995_2000	WY_CR	Synergy	Synergy	Synergy	Synergy
1995_2000	WY_LR	Trade-off	Trade-off	Trade-off	Trade-off
1995_2000	NPP_SC	Trade-off	Synergy	Synergy	Trade-off
1995_2000	NPP_HQ	Trade-off	Synergy	Synergy	Synergy
1995_2000	NPP_CF	Trade-off	Synergy	Synergy	Synergy
1995_2000	NPP_CR	Trade-off	Trade-off	Trade-off	Synergy
1995_2000	NPP_LR	Synergy	Trade-off	Trade-off	Synergy
1995_2000	SC_HQ	Trade-off	Trade-off	Synergy	Trade-off
1995_2000	SC_CF	Trade-off	Synergy	Synergy	Synergy
1995_2000	SC_CR	Trade-off	Trade-off	Trade-off	Synergy
1995_2000	SC_LR	Synergy	Trade-off	Synergy	Trade-off
1995_2000	HQ_CF	Trade-off	Synergy	Synergy	Trade-off
1995_2000	HQ_CR	Trade-off	Synergy	Synergy	Synergy
1995_2000	HQ_LR	Trade-off	Trade-off	Synergy	Trade-off
1995_2000	CF_CR	Trade-off	Trade-off	Trade-off	Trade-off
1995_2000	CF_LR	Synergy	Trade-off	Trade-off	Trade-off
1995_2000	CR_LR	Synergy	Trade-off	Trade-off	Synergy
2000_2005	WY_NPP	Trade-off	Synergy	Synergy	Trade-off
2000_2005	WY_SC	Trade-off	Synergy	Trade-off	Trade-off
2000_2005	WY_HQ	Trade-off	Trade-off	Trade-off	Trade-off
2000_2005	WY_CF	Trade-off	Synergy	Synergy	Synergy
2000_2005	WY_CR	Trade-off	Synergy	Synergy	Synergy
2000_2005	WY_LR	Trade-off	Trade-off	Trade-off	Trade-off
2000_2005	NPP_SC	Trade-off	Trade-off	Trade-off	Trade-off
2000_2005	NPP_HQ	Trade-off	Trade-off	Trade-off	Trade-off
2000_2005	NPP_CF	Trade-off	Synergy	Synergy	Synergy
2000_2005	NPP_CR	Trade-off	Trade-off	Trade-off	Trade-off
2000_2005	NPP_LR	Trade-off	Trade-off	Trade-off	Synergy
2000_2005	SC_HQ	Trade-off	Synergy	Synergy	Trade-off
2000_2005	SC_CF	Trade-off	Trade-off	Trade-off	Synergy
2000_2005	SC_CR	Trade-off	Synergy	Synergy	Synergy
2000_2005	SC_LR	Trade-off	Trade-off	Synergy	Trade-off
2000_2005	HQ_CF	Trade-off	Trade-off	Trade-off	Synergy
2000_2005	HQ_CR	Trade-off	Synergy	Synergy	Synergy
2000_2005	HQ_LR	Trade-off	Trade-off	Trade-off	Trade-off
2000_2005	CF_CR	Trade-off	Trade-off	Trade-off	Synergy

Period	Pair	TSS_type	Pearson_type	Spearman_type	Partial_type
2000_2005	CF_LR	Trade-off	Trade-off	Trade-off	Trade-off
2000_2005	CR_LR	Trade-off	Synergy	Synergy	Synergy
2005_2010	WY_NPP	Synergy	Synergy	Synergy	Synergy
2005_2010	WY_SC	Synergy	Synergy	Synergy	Synergy
2005_2010	WY_HQ	Synergy	Trade-off	Trade-off	Synergy
2005_2010	WY_CF	Synergy	Synergy	Synergy	Trade-off
2005_2010	WY_CR	Synergy	Synergy	Synergy	Synergy
2005_2010	WY_LR	Synergy	Trade-off	Trade-off	Trade-off
2005_2010	NPP_SC	Synergy	Trade-off	Trade-off	Synergy
2005_2010	NPP_HQ	Synergy	Trade-off	Trade-off	Synergy
2005_2010	NPP_CF	Synergy	Synergy	Synergy	Synergy
2005_2010	NPP_CR	Synergy	Synergy	Synergy	Trade-off
2005_2010	NPP_LR	Synergy	Trade-off	Trade-off	Trade-off
2005_2010	SC_HQ	Synergy	Trade-off	Trade-off	Trade-off
2005_2010	SC_CF	Synergy	Trade-off	Trade-off	Trade-off
2005_2010	SC_CR	Synergy	Synergy	Synergy	Trade-off
2005_2010	SC_LR	Synergy	Trade-off	Trade-off	Trade-off
2005_2010	HQ_CF	Synergy	Trade-off	Trade-off	Trade-off
2005_2010	HQ_CR	Synergy	Trade-off	Trade-off	Trade-off
2005_2010	HQ_LR	Synergy	Trade-off	Synergy	Trade-off
2005_2010	CF_CR	Synergy	Synergy	Synergy	Synergy
2005_2010	CF_LR	Synergy	Trade-off	Trade-off	Synergy
2005_2010	CR_LR	Synergy	Trade-off	Trade-off	Trade-off
2010_2015	WY_NPP	Trade-off	Trade-off	Trade-off	Trade-off
2010_2015	WY_SC	Trade-off	Synergy	Synergy	Synergy
2010_2015	WY_HQ	Trade-off	Trade-off	Synergy	Synergy
2010_2015	WY_CF	Trade-off	Trade-off	Trade-off	Synergy
2010_2015	WY_CR	Trade-off	Synergy	Synergy	Synergy
2010_2015	WY_LR	Trade-off	Trade-off	Trade-off	Trade-off
2010_2015	NPP_SC	Trade-off	Trade-off	Trade-off	Synergy
2010_2015	NPP_HQ	Trade-off	Trade-off	Trade-off	Trade-off
2010_2015	NPP_CF	Trade-off	Synergy	Synergy	Synergy
2010_2015	NPP_CR	Trade-off	Synergy	Synergy	Synergy
2010_2015	NPP_LR	Trade-off	Synergy	Synergy	Trade-off
2010_2015	SC_HQ	Trade-off	Trade-off	Trade-off	Synergy
2010_2015	SC_CF	Trade-off	Trade-off	Trade-off	Trade-off
2010_2015	SC_CR	Trade-off	Synergy	Synergy	Synergy
2010_2015	SC_LR	Trade-off	Synergy	Synergy	Synergy
2010_2015	HQ_CF	Trade-off	Trade-off	Trade-off	Synergy
2010_2015	HQ_CR	Trade-off	Trade-off	Trade-off	Trade-off
2010_2015	HQ_LR	Trade-off	Trade-off	Synergy	Trade-off
2010_2015	CF_CR	Trade-off	Synergy	Synergy	Trade-off
2010_2015	CF_LR	Trade-off	Synergy	Synergy	Synergy
2010_2015	CR_LR	Trade-off	Synergy	Synergy	Trade-off

Period	Pair	TSS_type	Pearson_type	Spearman_type	Partial_type
2015_2020	WY_NPP	Synergy	Synergy	Synergy	Synergy
2015_2020	WY_SC	Synergy	Synergy	Synergy	Synergy
2015_2020	WY_HQ	Synergy	Synergy	Synergy	Synergy
2015_2020	WY_CF	Synergy	Synergy	Synergy	Trade-off
2015_2020	WY_CR	Synergy	Synergy	Synergy	Synergy
2015_2020	WY_LR	Synergy	Synergy	Synergy	Trade-off
2015_2020	NPP_SC	Synergy	Trade-off	Trade-off	Synergy
2015_2020	NPP_HQ	Synergy	Synergy	Synergy	Synergy
2015_2020	NPP_CF	Synergy	Synergy	Synergy	Synergy
2015_2020	NPP_CR	Synergy	Synergy	Synergy	Trade-off
2015_2020	NPP_LR	Synergy	Synergy	Synergy	Trade-off
2015_2020	SC_HQ	Synergy	Synergy	Synergy	Synergy
2015_2020	SC_CF	Synergy	Trade-off	Trade-off	Trade-off
2015_2020	SC_CR	Synergy	Trade-off	Trade-off	Trade-off
2015_2020	SC_LR	Synergy	Synergy	Synergy	Synergy
2015_2020	HQ_CF	Synergy	Synergy	Synergy	Trade-off
2015_2020	HQ_CR	Synergy	Synergy	Synergy	Synergy
2015_2020	HQ_LR	Synergy	Trade-off	Synergy	Trade-off
2015_2020	CF_CR	Synergy	Synergy	Synergy	Synergy
2015_2020	CF_LR	Synergy	Synergy	Synergy	Synergy
2015_2020	CR_LR	Synergy	Synergy	Synergy	Synergy
2020_2024	WY_NPP	Synergy	Synergy	Synergy	Synergy
2020_2024	WY_SC	Synergy	Trade-off	Synergy	Synergy
2020_2024	WY_HQ	Synergy	Trade-off	Trade-off	Trade-off
2020_2024	WY_CF	Synergy	Synergy	Synergy	Trade-off
2020_2024	WY_CR	Synergy	Synergy	Synergy	Synergy
2020_2024	WY_LR	Synergy	Trade-off	Trade-off	Trade-off
2020_2024	NPP_SC	Synergy	Trade-off	Trade-off	Synergy
2020_2024	NPP_HQ	Synergy	Trade-off	Trade-off	Trade-off
2020_2024	NPP_CF	Synergy	Synergy	Synergy	Synergy
2020_2024	NPP_CR	Synergy	Synergy	Synergy	Trade-off
2020_2024	NPP_LR	Synergy	Synergy	Synergy	Trade-off
2020_2024	SC_HQ	Synergy	Trade-off	Trade-off	Trade-off
2020_2024	SC_CF	Synergy	Trade-off	Trade-off	Trade-off
2020_2024	SC_CR	Synergy	Trade-off	Trade-off	Trade-off
2020_2024	SC_LR	Synergy	Trade-off	Trade-off	Trade-off
2020_2024	HQ_CF	Synergy	Trade-off	Trade-off	Synergy
2020_2024	HQ_CR	Synergy	Synergy	Synergy	Synergy
2020_2024	HQ_LR	Synergy	Synergy	Synergy	Synergy
2020_2024	CF_CR	Synergy	Synergy	Synergy	Synergy
2020_2024	CF_LR	Synergy	Synergy	Synergy	Synergy
2020_2024	CR_LR	Synergy	Trade-off	Synergy	Synergy
1990_2024	WY_NPP	Synergy	Trade-off	Trade-off	Trade-off
1990_2024	WY_SC	Synergy	Trade-off	Trade-off	Trade-off
1990_2024	WY_HQ	Synergy	Synergy	Synergy	Synergy

Period	Pair	TSS_type	Pearson_type	Spearman_type	Partial_type
1990_2024	WY_CF	Synergy	Trade-off	Trade-off	Synergy
1990_2024	WY_CR	Trade-off	Synergy	Synergy	Synergy
1990_2024	WY_LR	Trade-off	Trade-off	Trade-off	Trade-off
1990_2024	NPP_SC	Trade-off	Trade-off	Trade-off	Trade-off
1990_2024	NPP_HQ	Trade-off	Trade-off	Trade-off	Trade-off
1990_2024	NPP_CF	Trade-off	Synergy	Synergy	Synergy
1990_2024	NPP_CR	Trade-off	Trade-off	Trade-off	Synergy
1990_2024	NPP_LR	Trade-off	Trade-off	Trade-off	Synergy
1990_2024	SC_HQ	Trade-off	Synergy	Synergy	Synergy
1990_2024	SC_CF	Trade-off	Trade-off	Trade-off	Synergy
1990_2024	SC_CR	Trade-off	Trade-off	Trade-off	Synergy
1990_2024	SC_LR	Trade-off	Trade-off	Trade-off	Trade-off
1990_2024	HQ_CF	Trade-off	Trade-off	Trade-off	Synergy
1990_2024	HQ_CR	Trade-off	Synergy	Trade-off	Trade-off
1990_2024	HQ_LR	Trade-off	Trade-off	Trade-off	Trade-off
1990_2024	CF_CR	Trade-off	Trade-off	Trade-off	Trade-off
1990_2024	CF_LR	Trade-off	Trade-off	Trade-off	Trade-off
1990_2024	CR_LR	Synergy	Trade-off	Trade-off	Trade-off

Table S 3. Period-level agreement between TSS and correlation-based classifications.

Period	Pearson consistency	Spearman consistency	Partial consistency
1990_1995	61.9047619047619	52.3809523809524	61.9047619047619
1990_2024	61.9047619047619	66.6666666666667	47.6190476190476
1995_2000	33.3333333333333	28.5714285714286	52.3809523809524
2000_2005	57.1428571428571	57.1428571428571	52.3809523809524
2005_2010	38.0952380952381	42.8571428571429	42.8571428571429
2010_2015	52.3809523809524	42.8571428571429	42.8571428571429
2015_2020	80.9523809523809	85.7142857142857	61.9047619047619
2020_2024	47.6190476190476	57.1428571428571	52.3809523809524

Table S 4. Sensitivity of TSS–ASI consistency under different absolute TSS thresholds.

threshold	agreement(%)	disagreement(%)
0	42.84	57.16
0.05	24.14	75.86
0.1	18.96	81.046
0.15	17.88	82.12
0.2	18.63	81.37

Table S 5. Sensitivity of dominant interaction patterns after excluding the CF–NPP pair.

period	Number of ES pairs after removing CF–NPP	Mean synergy ratio (%)	Mean trade-off ratio (%)
1990_1995	41	70.42	14.46
1995_2000	41	64.22	18.36
2000_2005	41	65.97	18.56
2005_2010	41	68.87	10.45
2010_2015	41	72.96	24.60
2015_2020	41	68.24	17.223
2020_2024	41	73.30	16.153
1990_2024	41	75.24	23.52

Table S 6 Bootstrap and leave-one-pair-out sensitivity analyses of TSS-derived interaction patterns

Period	Bootstrap mean agreement (%)	Bootstrap SD (%)	Stability index	Minimum synergy ratio after pair removal (%)	Maximum synergy ratio after pair removal (%)	Maximum deviation (%)
1990_1995	69.26	3.78	65.48801771	70.42	72.78	2.36
1995_2000	39.34	13.19	26.14554116	64.22	66.55	2.33
2000_2005	39.58	7.56	32.02223441	65.97	68.31	2.34
2005_2010	74.15	5.95	68.19599093	68.87	71.19	2.32
2010_2015	39.83	15.30	24.53031326	72.96	75.05	2.10
2015_2020	56.89	7.47	49.41965151	68.24	70.50	2.25
2020_2024	55.94	7.71	48.25371464	73.30	75.35	2.05
1990_2024	49.29	6.91	42.37679457	75.24	77.14	1.91

Table S 7. Detailed parameters and cross-validation performance of XGBoost models for predicting long-term TSS patterns.

Service pair	CV R^2	CV RMSE	eta	min_child_w eight	subsam ple	colsample_b ytree	lamb da	alp ha	Best boosting rounds
WY-SC	0.78	0.03	0.0<br>2	6.75	1.00	0.91	2.57	0.0<br>4	860.00
WY-CF	0.47	0.09	0.0<br>2	7.19	0.92	0.98	3.31	0.4<br>6	1190.00
WY-CR	0.74	0.05	0.0<br>1	4.21	0.66	0.69	1.50	0.6<br>9	1495.00
WY-LR	0.62	0.07	0.0<br>6	5.05	0.73	0.70	2.49	0.7<br>8	347.00
WY-<br>NPP	0.46	0.09	0.0<br>6	10.00	0.81	0.93	4.33	1.9<br>4	410.00
WY-HQ	0.20	0.13	0.0<br>1	2.85	0.72	0.79	3.97	0.8<br>7	1305.00

Service pair	CV R^2	CV RMSE	eta	min_child_weight	subsample	colsample_bytree	lambda	alpha	Best boosting rounds
SC-CF	0.58	0.04	0.08	5.13	0.74	0.99	0.53	0.55	418.00
SC-CR	0.87	0.01	0.03	8.88	0.90	0.84	1.65	0.04	860.00
SC-LR	0.67	0.02	0.01	9.41	0.86	0.95	0.92	0.07	1499.00
SC-NPP	0.58	0.04	0.07	3.52	0.67	0.91	2.58	0.14	857.00
SC-HQ	0.17	0.10	0.09	9.08	0.90	0.89	2.88	1.77	107.00
CF-CR	0.63	0.06	0.02	9.37	0.89	0.55	3.38	0.20	1431.00
CF-LR	0.48	0.07	0.09	1.62	0.95	0.90	2.10	0.72	325.00
CF-NPP	0.41	0.06	0.07	8.93	0.80	0.56	4.17	0.33	403.00
CF-HQ	0.13	0.15	0.02	4.71	0.87	0.64	1.62	1.14	552.00
CR-LR	0.76	0.03	0.02	6.10	0.75	0.70	4.28	0.11	1500.00
CR-NPP	0.63	0.06	0.07	6.12	0.93	0.71	4.18	0.13	324.00
CR-HQ	0.26	0.13	0.00	1.44	0.99	0.79	0.10	1.24	1500.00
LR-NPP	0.48	0.07	0.03	3.15	0.73	0.87	1.24	1.84	1498.00
LR-HQ	0.20	0.13	0.04	4.80	0.99	0.81	2.86	1.71	262.00
NPP-HQ	0.13	0.15	0.04	9.13	0.96	0.89	0.69	0.03	212.00

4 Supplementary Figures

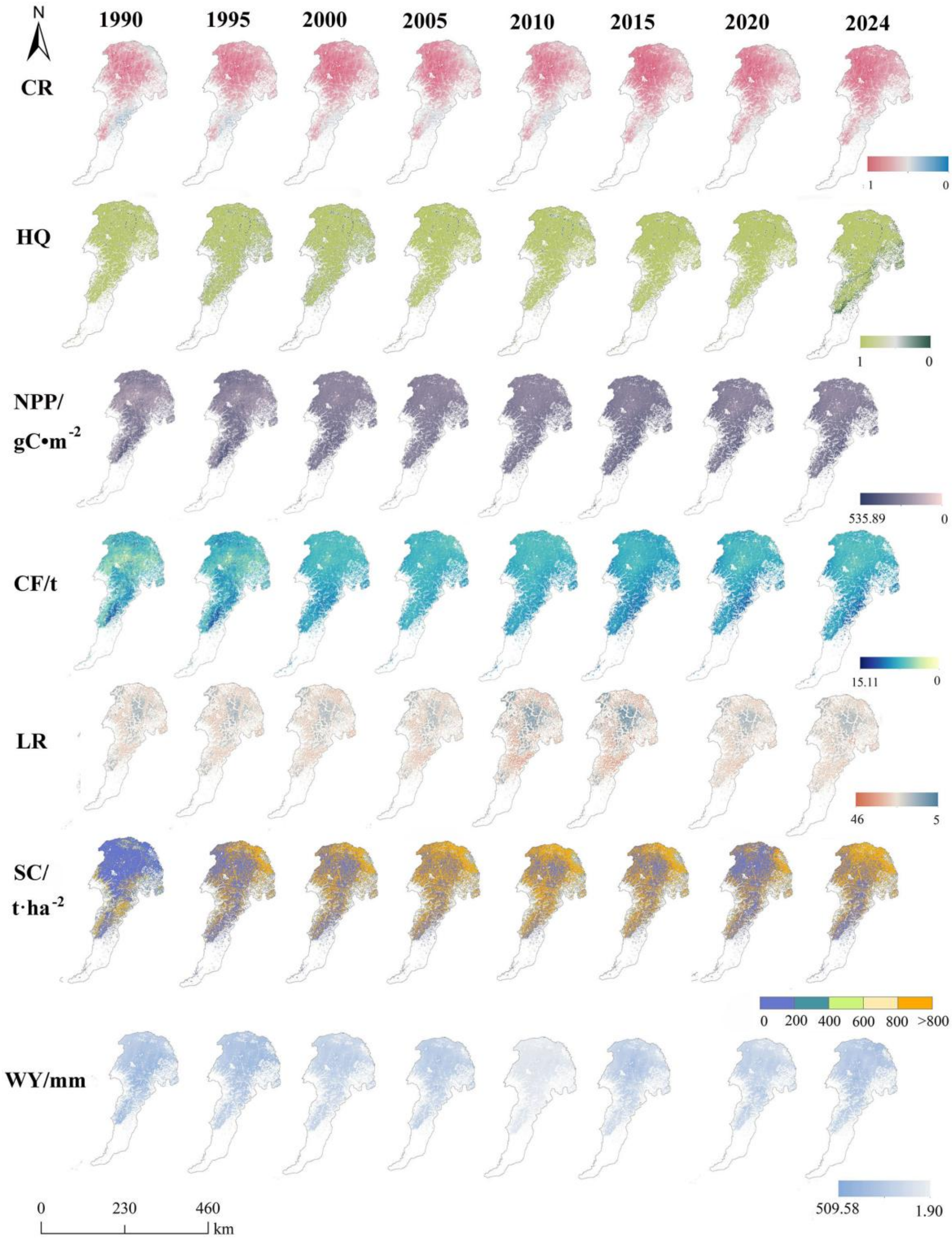

Figure S 1. Spatial patterns of the forest ecosystem services in Great Khingan Mountains between 1990 and 2024.

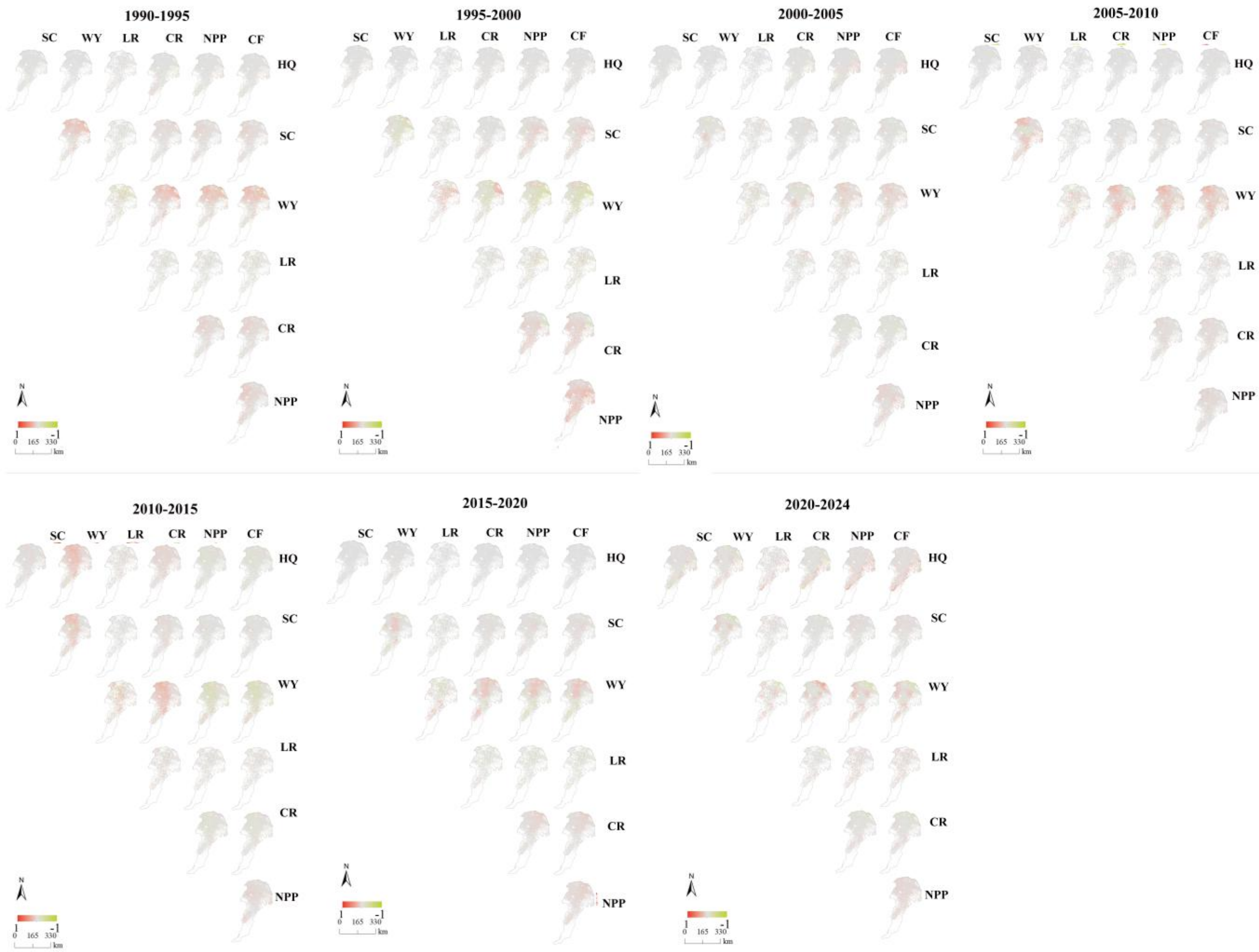

Figure S 2. Spatial patterns of the trade-off synergy strength in Great Khingan Mountains in 7 periods.

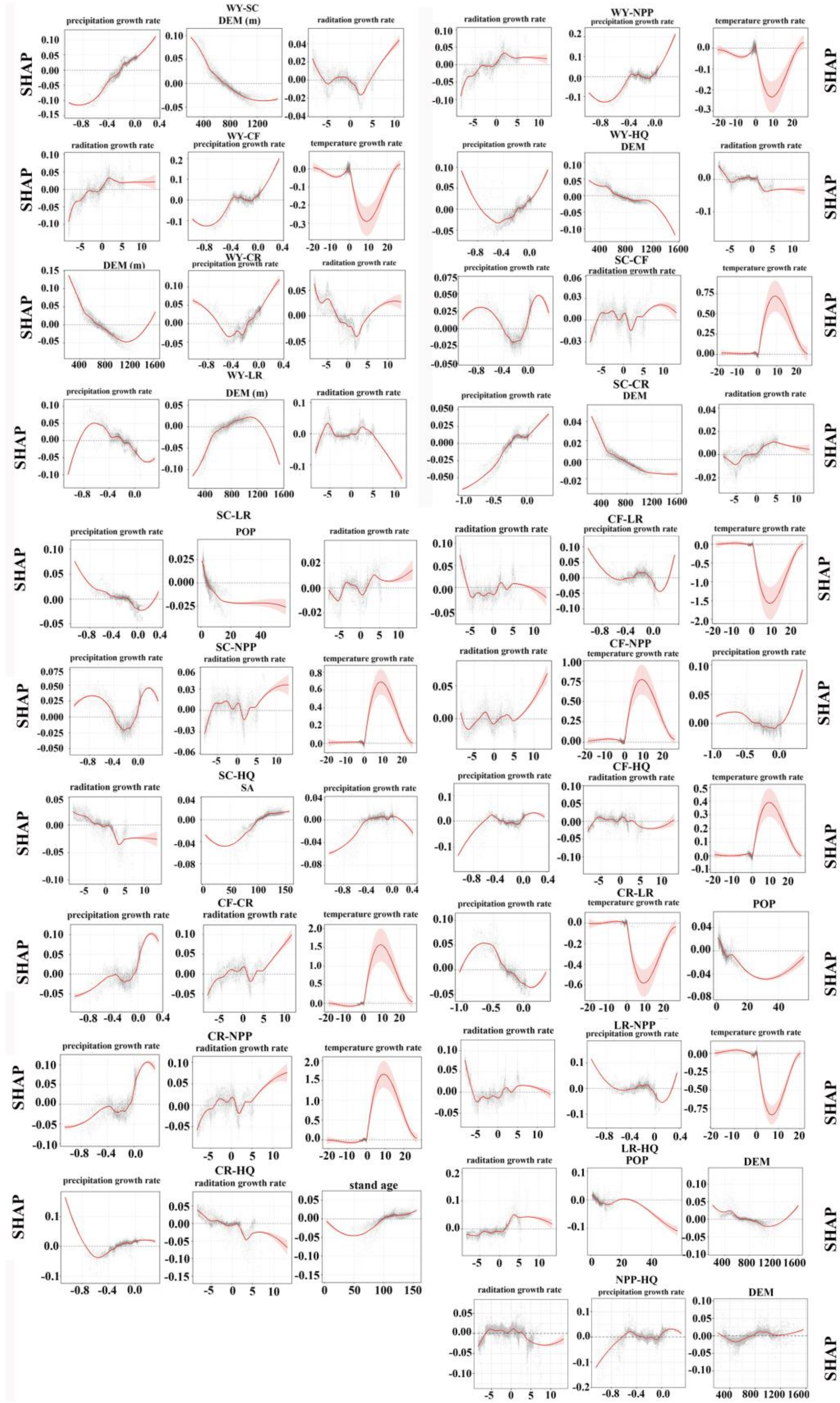

Figure S 3. SHAP dependence plots showing the response patterns of environmental drivers for different TSS relationships.

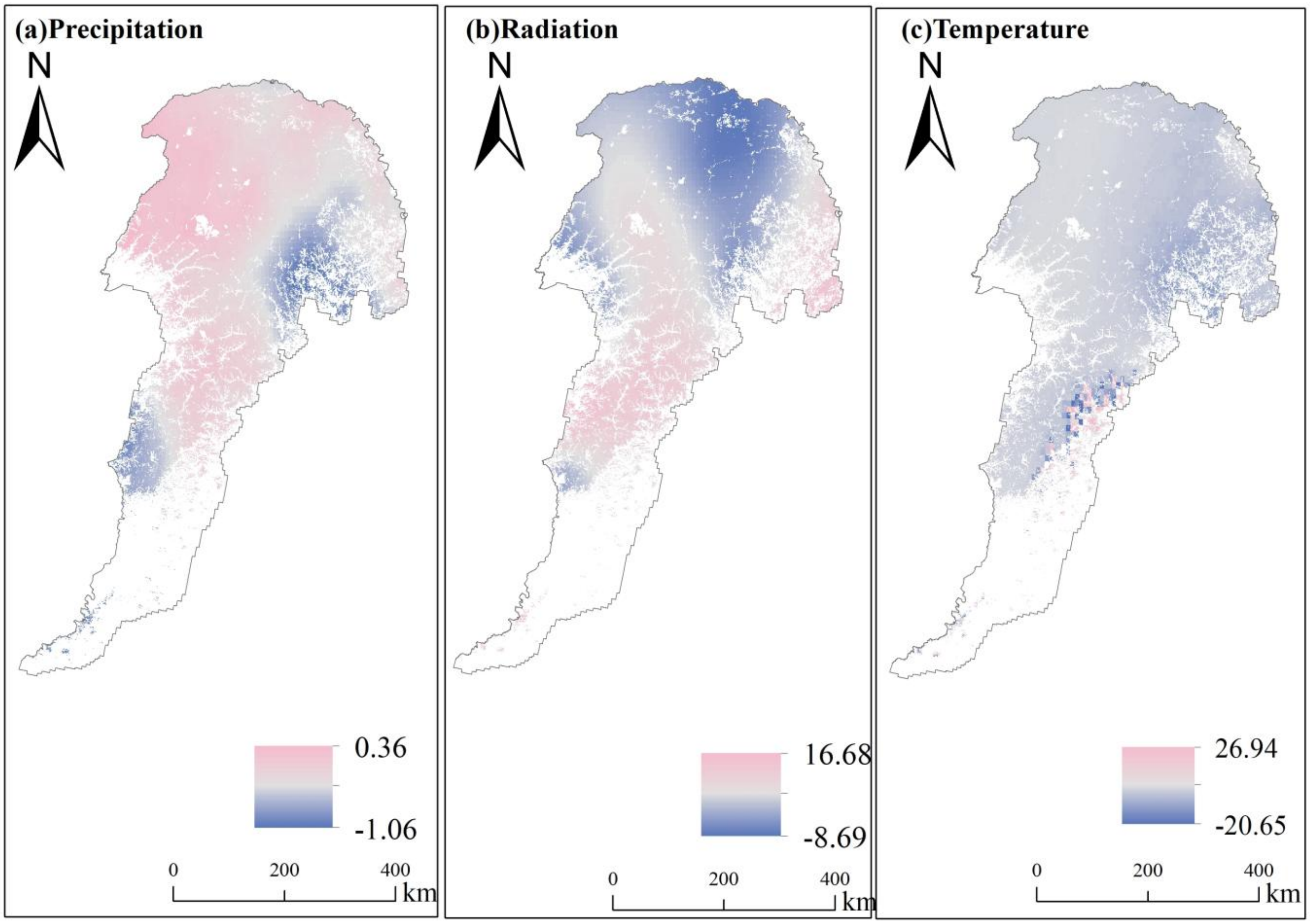

Figure S 4. Slope of meteorological factor variation.

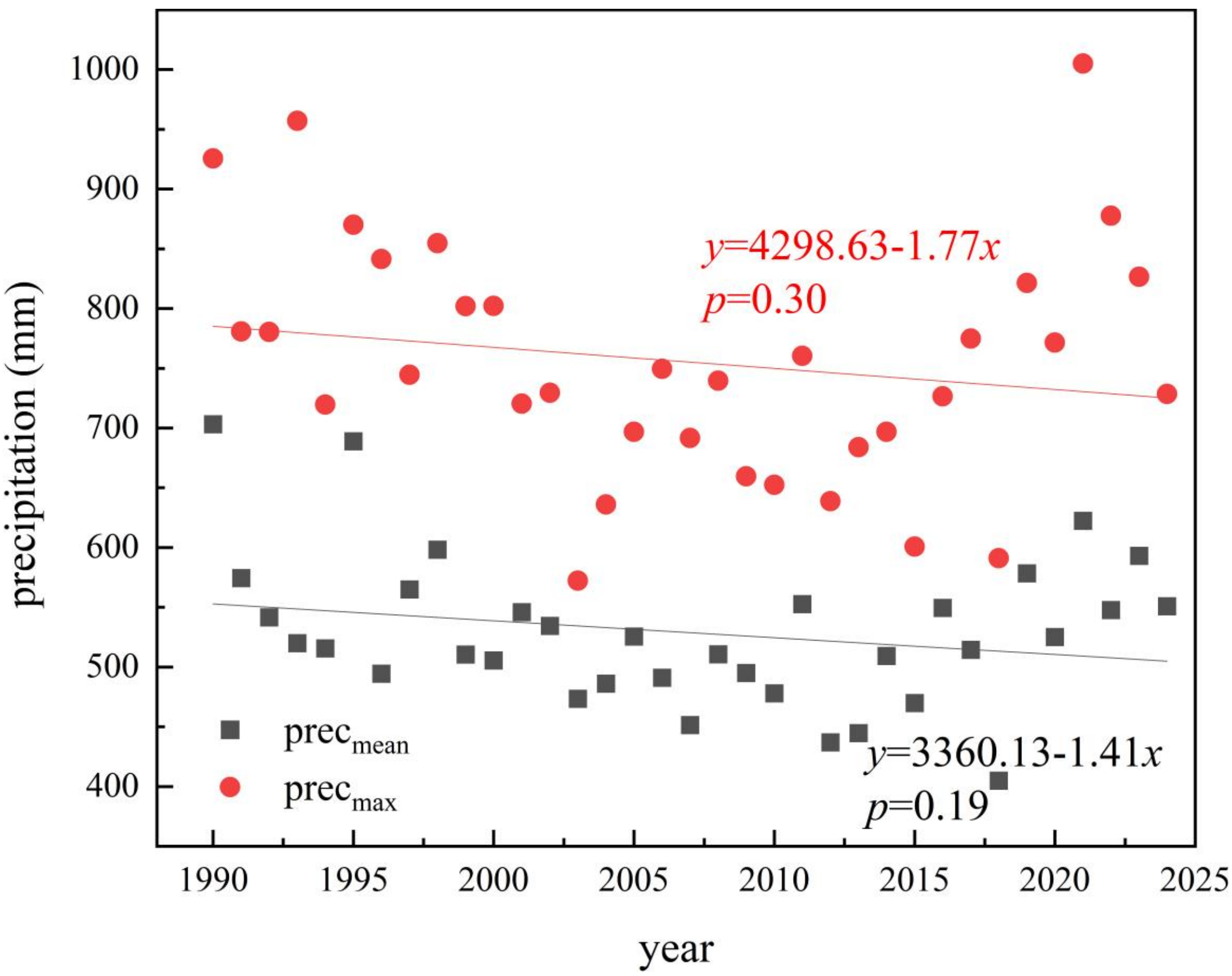

Figure S 5. The precipitation variation curve of Great Khingan Mountains from 1990 to 2024.

References

Li, B., Chen, D., Wu, S., Zhou, S., Wang, T., Chen, H., 2016. Spatio-temporal assessment of urbanization impacts on ecosystem services: Case study of Nanjing City, China. Ecological Indicators 71, 416–427. https://doi.org/10.1016/j.ecolind.2016.07.017

Li, Z., Jia, W., Li, T., Zhao, G., 2025. Dynamic modeling of effective crown height in *larix olgensis*: a synergistic analysis of stand density, height diameter ratio, and climatic factors. Forest Ecology and Management 598, 123210. https://doi.org/10.1016/j.foreco.2025.123210

Lopezosa, P., Berdugo, M., Morales-Márquez, J., Pastor, E., Delgado-Baquerizo, M., Bonet, A., Wang, J., Singh, B.K., Soliveres, S., 2023. On the relative importance of resource availability and habitat connectivity as drivers of soil biodiversity in Mediterranean ecosystems. Journal of Ecology 111, 1455–1467. https://doi.org/10.1111/1365-2745.14106

Paracchini, M.L., Zulian, G., Kopperoinen, L., Maes, J., Schägner, J.P., Termansen, M., Zandersen, M., Perez-Soba, M., Scholefield, P.A., Bidoglio, G., 2014. Mapping cultural ecosystem services: A framework to assess the potential for outdoor recreation across the EU. Ecological Indicators 45, 371–385. https://doi.org/10.1016/j.ecolind.2014.04.018

Shang, Y. (2024). Identification of ecological restoration areas and restoration strategies in Western Jilin Province. Doctoral dissertation, Jilin University, Changchun, China. https://doi.org/10.27162/d.cnki.gjlin.2024.000325

Shifaw, E., Sha, J., Li, X., Zhao, P., Ji, J., Zhang, H., Kassaye, A.Y., Lai, S., Ji, Z., Yang, Y., 2025. An innovative approach to prioritizing soil conservation areas under diverse scenarios by leveraging the complementary roles of soil retention services and soil erosion indicators. Journal of Hydrology: Regional Studies 57, 102102. https://doi.org/10.1016/j.ejrh.2024.102102

Wang, Y., Ye, A., Peng, D., Miao, C., Di, Z., Gong, W., 2022. Spatiotemporal variations in water conservation function of the Tibetan Plateau under climate change based on InVEST model. Journal of Hydrology: Regional Studies 41, 101064. https://doi.org/10.1016/j.ejrh.2022.101064

Wu, L., Sun, C., Fan, F., Wu, L., Sun, C., Fan, F., 2021. Estimating the Characteristic Spatiotemporal Variation in Habitat Quality Using the InVEST Model—A Case Study from Guangdong–Hong Kong–Macao Greater Bay Area. Remote Sensing 13. https://doi.org/10.3390/rs13051008
